# Advances in pH-Responsive Release Technologies in Food System: Mechanisms, Strategies, Application Forms and Future Directions

**DOI:** 10.3390/foods14223896

**Published:** 2025-11-14

**Authors:** Lidan Zhang, Junjun Zhang, Jianing Zhang, Xiaowei Huang, Jiyong Shi

**Affiliations:** Agricultural Product Processing and Storage Lab, School of Food and Biological Engineering, Jiangsu University, Zhenjiang 212013, China

**Keywords:** pH-responsive, controlled release mechanisms, design strategies, polymer, food preservation

## Abstract

pH-responsive technology enables precise control over the release of functional molecules, thereby maximizing their bioavailability. As the first comprehensive assessment of pH-responsive systems within food science, this review systematically examines the mechanism of pH-triggered release, which covers the protonation and deprotonation of functional groups and the breaking of dynamic covalent bonds (such as imines, disulfides, and metal coordination bonds). The design strategies, responsiveness, and application potential of key carrier materials are evaluated. In addition, the applications of pH-responsive release technologies in nutrient delivery, flavor encapsulation, and food preservation are highlighted, demonstrating enhanced bioavailability, extended shelf life, and improved sensory quality. Despite promising advancements achieved so far, significant challenges remain in ensuring material stability, and meeting safety and regulatory requirements. Future research directions are proposed, including the development of food-grade, eco-friendly, and stable carrier materials, the leveraging of AI-driven optimization for integrated systems, integrating multi-stimuli responsiveness, and establishing robust safety profiles to facilitate regulatory approval, collectively establishing a solid foundation for next-generation sustainable and intelligent food packaging and delivery systems.

## 1. Introduction

### 1.1. Background and Significance

As consumers place higher demands on areas such as food preservation, nutritional enhancement, and flavor fortification, the critical role of functional molecules, including preservatives, nutrients, and essential oils, has become increasingly prominent. However, inherent limitations of these molecules, such as low bioavailability and susceptibility to inactivation during processing and storage, have increasingly become major obstacles hindering their effective application [1,2,3].

These challenges permeate several critical domains of food development. In the area of food preservation, microbial contamination remains a persistent issue, leading to substantial economic losses and public health concerns [4,5]. Although active packaging has been widely implemented, the premature release, volatility, or degradation of active compounds during processing and storage, along with potential misuse, have raised questions regarding its reliability and safety [6,7]. In the field of nutritional fortification, a significant challenge is the low bioavailability of many bioactive compounds. Sensitive ingredients often degrade in the stomach due to harsh gastric conditions. As a result, they may not reach the intestinal absorption sites effectively [8,9]. Likewise, the volatility and chemical instability of key aroma compounds during processing and storage lead to significant flavor loss, undermining product appeal [10].

Conventional encapsulation approaches, such as those employed for essential oils [11,12], and anthocyanins [13], also faced such challenges. This gap underscores the urgent need for smarter delivery systems that can respond to environmental triggers, protecting ingredients during storage and releasing them on demand. Typical stimuli exploited for such purposes include pH [14], enzymes [15,16,17], humidity [18], light [19], temperature [20], and glucose [21]. Among these, pH-responsive release is particularly promising due to the well-defined pH gradients present throughout food processing, storage, and gastrointestinal transit. This technology, defined as the integration of active compounds with pH-sensitive carriers to enable controlled release [3], it has found extensive applications from pharmaceuticals to agriculture [22,23,24].

Within food science, pH-controlled release technology holds significant promise for intelligent antibacterial packaging, nutrient delivery, and flavor enhancement, contributing to extended shelf life, improved sensory quality, and ultimately, food safety and public health. For instance, Feng et al. and Su et al. developed advanced curcumin delivery systems that achieved controlled intestinal release and significantly enhanced bioavailability, stability, and bioaccessibility [25,26]. Similarly, in food preservation, gelatin films embedded with eugenol-loaded nanoparticles exhibited acid-responsive release and effectively extended food shelf life without compromising quality, demonstrating significant potential for active packaging applications [4].

These advanced systems are fabricated using different encapsulation platforms, including liposomes [27,28], microcapsules [29,30], nanoparticles [31], and Pickering emulsions [31]. Furthermore, emerging technologies such as ultrasonication [32,33], electrohydrodynamic processes [34], pH-driven assembly [35,36], and electrospinning [5,17] have been adopted to improve encapsulation efficiency and functional performance. The core of the functional realization of these controlled release systems lies in their carrier materials and response mechanisms. These carriers are often made from polymeric or nanostructured materials. They allow controlled release through simple mechanisms, such as structural changes caused by protonation or deprotonation, or the breaking of dynamic covalent bonds under pH changes. Release behavior can also be studied with mathematical models that describe and analyze release kinetics.

### 1.2. Challenges and Opportunities

Although these packaging technologies and materials have made significant progress, laying a solid foundation for pH-responsive release systems in smart packaging, the technology still faces a series of challenges in its practical application. Research efforts focus on improving sensitivity, specificity, scalability, and material safety. In order to overcome these challenges, future research will focus on identifying sustainable materials, integrating multi-responsive systems, and applying AI technologies for comprehensive optimization [37].

Although there has been substantial research on pH-controlled release technology, a comprehensive review that systematically introduces its mechanisms, design strategies, carrier systems, and applications in the food industry is still lacking. This review aims to fill that gap by providing a detailed discussion of the controlled release mechanisms, carrier materials, and release models related to pH-responsive systems.

Relevant studies published from 2020 onward were retrieved from Elsevier, Web of Science, and Google Scholar using keywords such as ‘pH sensitive’, ‘mechanism’, ‘flavor’, ‘nutrient delivery’, and ‘controlled release polymer’. The inclusion criteria encompassed pH-responsive delivery systems, primarily targeting studies on the release of food-active ingredients (e.g., nutrients, flavors) in food matrices, simulants, or gastrointestinal environments. To address the scarcity of research on controlled release mechanisms in food science, we additionally incorporated pertinent non-food research that elucidated fundamental release mechanisms (e.g., protonation/deprotonation, dynamic covalent bond cleavage). We excluded non-English/Chinese publications, non-original research, patents, conference abstracts, and studies with incomplete data or an irrelevant focus. From an initial 337 records, 161 met the eligibility criteria and were included in this review.

## 2. Mechanism of pH-Triggered Release

### 2.1. Overview of pH-Triggered Release Scenarios

pH-responsive smart packaging enables the precise release of active compounds by detecting pH changes caused by food spoilage or physiological environments. Controlled release systems based on pH changes can be broadly categorized into two types according to the direction of the pH shift.

#### 2.1.1. Release Mechanisms and Applications Triggered by pH Reduction

This mechanism is primarily initiated by the generation of acidic substances. In the field of food preservation, the respiration of fresh produce, along with microbial activity, produces carbon dioxide (CO_2_), which dissolves in water to form carbonic acid [38]. Concurrently, microorganisms such as bacteria directly secrete various organic acids [39]. These processes lead to a decrease in the pH of the microenvironment.

#### 2.1.2. Release Mechanisms and Applications Triggered by pH Elevation

This mechanism mainly originates from the production of alkaline substances. In food preservation, it is particularly relevant for protein-rich products such as meat and fish, where microbial spoilage and protein deamination generate volatile basic nitrogenous compounds, such as ammonia, dimethylamine, and trimethylamine, leading to a pH increase [40]. In nutrient delivery, this mechanism is designed for targeted intestinal release. Carrier materials can resist the strongly acidic gastric environment and then undergo deprotonation, erosion, or structural disintegration in the near-neutral to weakly alkaline pH of the intestine, enabling the precise release of encapsulated nutrients [41].

### 2.2. Overview of the Driving Forces of pH-Responsive Systems

The release of active agents is governed by a combination of physical mass transfer processes, chemical triggers, and bio-activated release mechanisms. Based on the driving forces, the release mechanisms can be categorized into the following types.

#### 2.2.1. Fundamental Release Mechanisms Governed by Physical Processes

(1) Diffusion-induced release refers to the process in which active agents diffuse through the microporous or macroporous structure of a polymer and are delivered from the surface of the film into the food. The chemical properties, porosity, and permeability of the polymer are the key parameters influencing the release rate in this type of mechanism [42]. For controlled-release systems comprising polymers such as PCL, PLA, and L100, the initial release of hydrophobic components (e.g., essential oils) is primarily governed by a diffusion-mediated mechanism.
(2) Swelling-induced release: The low diffusion coefficient of the antimicrobial agent reduces its diffusion rate within the polymer. When the polymer is placed in a compatible liquid medium, the liquid penetrates the polymer matrix and causes swelling. In the swollen state, the diffusion coefficient of the active agent increases, resulting in a higher release rate. Since most foods are moist, water is the most common penetrating medium. Therefore, this type of release often occurs in moisture-sensitive packaging materials such as protein or polysaccharide-based films [42]. Hydrogels, as three-dimensional cross-linked networks capable of absorbing large amounts of water without dissolving, further extend this concept with environmental responsiveness. The sensitivity of free radical reactions to pH is, in itself, critically moderated by the water content of the food matrix. In high-moisture environments, the high mobility of H^+^ and OH^−^ ions ensures that any pH shift is rapidly transmitted throughout the system [43].
(3) Disintegration-induced release is primarily caused by the degradation, cleavage, or deformation of the polymer, which results from changes in the fluid properties of the polymer matrix. This type of release can occur in certain polymers, such as poly (anhydride), poly (lactide), and poly (lactide–*co*–glycolide), which are characterized by targeted release properties [42].

#### 2.2.2. Advanced Responsive Mechanisms: Chemical Triggers and Bio-Activated Release

(1) Protonation or deprotonation of ionizable groups regulates electrostatic interactions, which cause the polyelectrolytes to expand or contract. This process speeds up or slows down the release. The carboxyl group (–COOH) protonates under acidic conditions, promoting contraction; under alkaline conditions, it undergoes deprotonation to hydrophilic –COO^−^, causing swelling and release. In contrast, the amino group (–NH_2_) protonates to –NH_3_^+^ under acidic conditions, promoting the release. At higher pH values, diffusion is limited due to deprotonation contraction. This mechanism has been demonstrated in carboxymethyl chitosan/alginate hydrogels, which can achieve controlled release [44].
(2) pH-dependent antioxidant activation and release: The radical-scavenging activity of many potent phenolic antioxidants (e.g., ferulic acid, quercetin) is intrinsically pH-dependent, governed by the acid dissociation constant (pKa) of their phenolic hydroxyl groups. In acidic solutions, the molecular form dominates, while in alkaline mediums (pH > pKa), the anionic form (phenolate anions) is the predominant species [45]. The phenolic hydroxyl group (-OH) can undergo pH-dependent deprotonation, and the resulting phenolate anion can inactivate free radicals via fast electron transfer (sequential proton loss electron transfer mechanism) [46].
(3) Free radical-induced degradation as a release trigger: This mechanism describes an intelligent, responsive process where the packaging system is designed to detect and counteract free radicals generated within the food during spoilage. When a packaging system is exposed to pro-oxidative conditions (e.g., light, heat, metal ions), a radical chain reaction can be initiated in the food, generating lipid peroxyl radicals (LOO•) [47,48]. These LOO• radicals act as key chain carriers, propagating the cycle by abstracting hydrogen from lipids to form lipid hydroperoxides (LOOH), which subsequently decompose into volatile off-flavors and additional radicals. For instance, the formation rate of hydroxyl radicals (•OH) is typically higher under acidic to neutral conditions compared to alkaline environments. At elevated pH, hydrogen peroxide (H_2_O_2_) is more prone to decomposition, which diminishes its availability for producing reactive oxygen species (ROS). Additionally, the overall reaction kinetics can be hindered at higher pH levels [49]. Packaging systems can be engineered to incorporate antioxidants that interrupt this process by serving as chain-breaking agents, quenching propagating radicals, and thereby terminating the destructive chain reaction [47,48].

In summary, release is governed by the following distinct mechanisms: diffusion-induced release is driven by concentration gradients; swelling-induced release is dominated by liquid absorption and polymer expansion; disintegration-induced release is caused by polymer degradation; and pH-dependent release is regulated by ionic interactions that directly alter polymer solubility and chain conformation. Beyond these, sophisticated chemical mechanisms, such as pH-dependent antioxidant activation and free radical-induced degradation, enable a more intelligent, responsive, and efficient release profile, directly linking the release trigger to the spoilage chemistry itself. These mechanisms differ in their driving forces, controlling parameters, and material suitability, offering diverse strategies for designing active or controlled-release packaging systems.

## 3. Design Strategies and Carrier Materials

### 3.1. An Overview of Acid-Sensitive Covalent Bonding

#### 3.1.1. Imine Bond

The pH-responsive behaviors of various acid-sensitive covalent bonds are summarized in Table 1. Imine bonds, formed through Schiff-base reactions between amine and aldehyde groups [50], exhibit pH-responsive reversibility due to their dynamic covalent nature. The primary advantage of imine bonds is their pH-responsive reversibility. However, this is also their main limitation; they are susceptible to acid-catalyzed hydrolysis. Under acidic conditions, the hydrolysis of imine bonds releases active compounds, while neutral or alkaline environments stabilize the bonds, enabling controlled release [51]. This mechanism is critical in food packaging, where spoilage-induced pH changes trigger antimicrobial agent release. For instance, imine bonds were synthesized between the primary amine groups of chitosan and the carbonyl groups of antifungal aldehydes, and the resulting functionalized chitosan films demonstrated pH-dependent hydrolysis, releasing the perillaldehyde under pH four conditions, effectively extending the shelf-life of berries from 3 to 12 days [52]. Similarly, cinnamaldehyde (CA) encapsulated in chitosan-fucoidan films via imine bonds showed accelerated release at pH 5, achieving 2.3-fold higher antibacterial activity compared to pH 7, thereby extending litchi shelf life [53].

**Figure 1:**
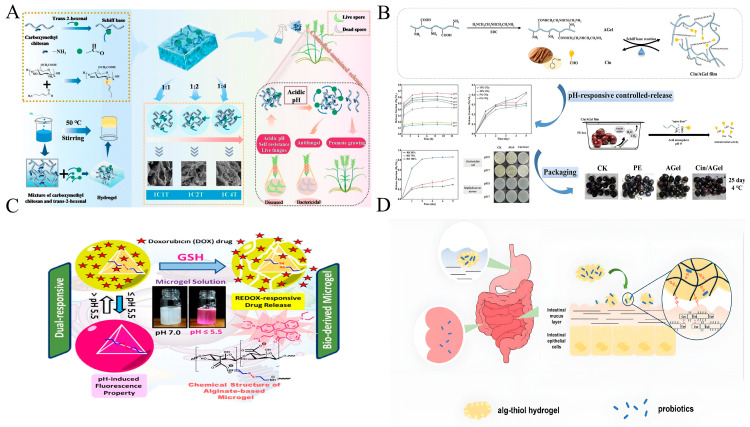
Acid-responsive mechanism diagram of imine bonds and disulfide bonds. (**A**) pH-responsive release through dynamic Schiff-base bonds [54], (**B**) cinnamaldehyde/aminated gelatin film as pH-responsive controlled-release packaging [55], (**C**) pH/Redox-responsive disulfide alginate microgels for anticancer drug delivery [60], (**D**) disulfide bond-mediated pH/Redox dual-responsive release [58].

The density of imine bonds can be adjusted, which increases application flexibility. Zhou et al. (Figure 1A) [54] prepared carboxymethyl chitosan (CMCS)-T2H hydrogels with tunable crosslinking by changing the aldehyde content. They released T2H in response to pathogen-induced acidity, which provided sustained antifungal protection for crops. In cherry preservation, Tang et al. (Figure 1B) [55] designed cinnamaldehyde/aminated gelatin (Cin/AGel) films that used imine bonds to release cinnamaldehyde under acidification caused by CO_2_ and humidity. These films extended the shelf life of cherries by 9 days at 4 °C. Such systems highlight the dual functionality of imine bonds: stabilizing active agents during storage and enabling precise release during spoilage.

Moreover, imine-based materials also show self-healing properties. For instance, dual-network hydrogels that combine imine and borate ester bonds were able to repair themselves at room temperature, which preserved structural integrity while delivering antibacterial agents [56]. This property shows the potential of imine bonds for creating strong and responsive packaging that can adjust to changing food microenvironments. Challenges in improving bond stability and controlling release kinetics remain, and ongoing studies continue to address these issues [52,57].

#### 3.1.2. Disulfide Bond

Disulfide bonds (S–S) are formed by the oxidation of sulfhydryl groups. These bonds can be cleaved under reducing conditions that are biologically relevant. Due to their dynamic nature, disulfide bonds are often combined with pH-sensitive groups to create dual-responsive systems for controlled release applications. A representative example is the disulfide-crosslinked thiolated alginate hydrogel, which effectively enhances probiotic survival through a pH and redox-responsive release mechanism. In the acidic gastric environment, protonation induces hydrogel contraction, thereby shielding encapsulated probiotics. Upon entering the small intestine, deprotonation of carboxyl groups prompts hydrogel swelling and initiates partial probiotic release. Finally, upon reaching the colon, reductive cleavage of disulfide bonds by glutathione leads to complete hydrogel degradation and the subsequent release of remaining probiotics (Figure 1D) [58]. Similarly, pH/redox dual-responsive micelles based on mPEG-imine-SS-PCL remain stable at physiological pH but rapidly release doxorubicin under tumor microenvironment conditions (pH 5.5, high GSH), greatly improving antitumor efficacy [59]. This dual responsiveness is further exemplified by a microgel system fabricated from disulfide-crosslinked sodium alginate, which enables redox-triggered drug release under reducing conditions while providing pH-dependent fluorescence tracking capability, demonstrating considerable potential for targeted cancer therapy (Figure 1C) [60].

#### 3.1.3. Metal Coordination Bond

Metal coordination bonds form stable but reversible structures through interactions between metal ions and electron-donating ligands. These bonds are highly sensitive to pH changes, as variations in proton concentration can alter bond strength. This leads to dissociation and controlled release of encapsulated agents, making metal coordination bonds ideal for stimuli-responsive applications [61], such as targeted drug delivery and active packaging.

**Figure 2:**
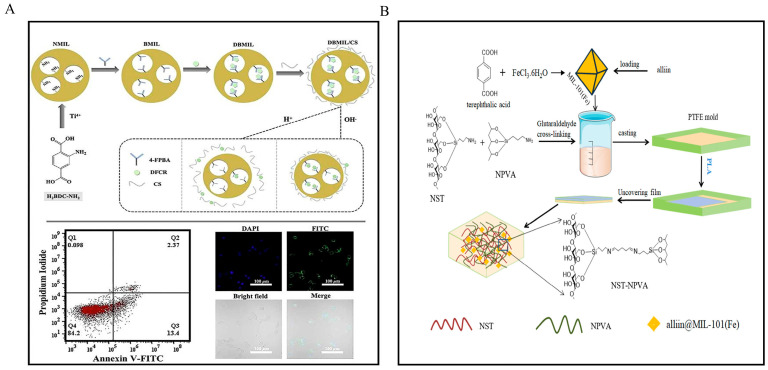
pH response mechanism diagram of the metal coordination bond. (**A**) pH-responsive drug release through metal–organic coordination [62], (**B**) MIL-101(Fe) carrier for pH-responsive release [63].

Metal–organic frameworks (MOFs) use pH-sensitive coordination bonds to control drug release. This approach reduces systemic toxicity and enhances therapeutic effectiveness (Figure 2A) [62]. Similarly, metal-phenolic networks (MPNs) are effective nanocarriers through the acidic environment in tumors to trigger drug release [61]. Furthermore, MIL-101(Fe) framework loaded with alliin releases the compound more quickly under acidic conditions due to the instability of Fe-carboxylate bonds. This feature helps extend the shelf life of strawberries through antimicrobial packaging (Figure 2B) [63].

In addition to affecting release behavior, metal coordination can improve material properties. For example, adding Fe^3+^-pyridine coordination to epoxidized natural rubber improves its mechanical strength and self-healing ability [64]. These platforms improve the bioavailability of bioactive compounds, making them effective carriers for controlled release.

#### 3.1.4. Acylhydrazone

Acylhydrazone bonds (–C=N–NH–) are a reliable mechanism for pH-responsive drug delivery due to their hydrolysis under acidic conditions. These bonds are stable at physiological pH but break down quickly in acidic environments. Under acidic conditions, it is susceptible to nucleophilic attack by water molecules, leading to the hydrolysis. This hydrolysis allows for the controlled release of conjugated drugs, as seen with doxorubicin (DOX) loaded systems.

For instance, SPIO nanocomposites loaded with DOX through acylhydrazone linkages much faster drug release at pH 5.0 than at pH 7.4, promoting tumor-specific delivery [65]. Similarly, amine-functionalized mesoporous silica nanoparticles (NH_2_-MSNs) were linked to oxPL through an acylhydrazone bond to create a gated system that loaded 5-fluorouracil. Under acidic conditions, this bond hydrolyzed, breaking the oxPL coating and releasing the encapsulated 5-FU [66].

These systems utilize the acidic tumor microenvironment (TME) to control drug release. Pectin-PEG hydrogels, crosslinked by acylhydrazone bonds, degrade at low pH. This selective degradation leads to higher DOX release in CT-26 tumors, improving antitumor effects while reducing side effects [67]. Similarly, pH-sensitive DHPD nanoparticles efficiently cleave acylhydrazone bonds in the acidic TME. This process enhances DOX delivery to breast cancer cells and promotes antitumor immunity by increasing cytokine levels, such as IFN-γ and TNF-α [68].

Recent advances incorporate acylhydrazone chemistry into multifunctional platforms for improved performance. Nanocellulose hydrogels with dual dynamic bonds, including borate ester bond and acylhydrazone bond, achieved a high self-healing efficiency of 92.7% and better mechanical flexibility [69]. Similarly, the incorporation of dual pH-sensitive dynamic covalent bonds (acylhydrazone and imine bonds) into chitosan/konjac glucomannan hydrogels creates a dynamic network with self-healing and injectable capabilities, making it a promising platform for the controlled and sustained release of protein drugs [70]. These designs demonstrate the potential of the acylhydrazone bond for drug release in advanced therapeutic applications.

#### 3.1.5. Boronate Ester Bond

Boronate ester bonds (B–O) form through reversible interactions between boric acids and diols, exhibiting pH-dependent stability. They remain stable in neutral or alkaline conditions, but hydrolyze in acidic environments. This process enables precise pH-responsive control in drug delivery applications.

In drug delivery, boronate-based systems enable targeted release under acidic conditions. Ren et al. developed boronated cyclodextrin frameworks (BCF) for pulmonary delivery of extracellular vesicles. These frameworks achieved triggered release in inflammatory lung tissues through pH/H_2_O_2_ response [71]. Similarly, Chen et al. [72] designed boronate-linked micelles that synergistically release doxorubicin and methotrexate in cancer cells. Additionally, Kim et al. [73] utilize the reduction in electrical resistance caused by cleavage of the boronate ester bond to detect tumor acidity, enabling real-time monitoring.

Recent advances combine boronate chemistry with other stimuli for enhanced functionality. Wang et al. [74] built pH/ROS dual-responsive nanogels through boronate crosslinking. These nanogels supported combined chemoimmunotherapy. Liang et al. [75] created chitosan-based hydrogels with dual dynamic bonds, such as boronate and Schiff bonds, for infected wound treatment, demonstrating improved biocompatibility and antibacterial performance.

**Table 1:** pH-responsive behavior of acid-sensitive covalent bonds.

Mechanism	Application Forms	Response Range	Release Behavior	Application Effects	Reference
Schiff-based imine bond	hydrogel	pH 3, 5, 7	At pH 3 and pH 5, the maximum concentration of T2H from 1C1T was 7.83 mg/mL and 7.23 mg/mL after 12 h. While at pH 7.0, that of T2H was 1.75 mg/mL after 24 h.	offering sustained antifungal protection for crops	[54]
imine bond	biopolymer	pH 4, 7	The effectiveness of CSHX films against B. cinerea was fungicidal at pH 4 and inhibitory at pH 7.	effectively extending the shelf-life of berries from 3 to 12 days	[52]
imine bond	film	pH 1–7	Cinnamaldehyde is released slowly in neutral solutions (pH 6–7), and release increases at pH 5. Higher cinnamaldehyde release in a pH 1–4 solution.	extending the shelf life of cherries by 9 days at 4 °C	[55]
disulfide bond	hydrogel	pH 2.0, 7.4	Under alkaline conditions, deprotonation of –COOH groups results in the pH-responsive release of probiotics. On the other hand, the acidic condition causes the shrinkage of the hydrogel.	The hydrogel achieved targeted probiotic release under neutral and alkaline conditions.	[58]
disulfide bond	nanogel	pH 5.5, 7.4	DTX release from NGs at pH 7.4 plateaus at 11% after 2 h. An acidic environment (pH 5.5) boosts the release compared to the release in 10 mM GSH alone.	Nanogels were loaded with a administered anticancer drug, docetaxel,	[76]
the imine and disulfide bonds	micelle	pH 5.5, 7.4	At pH = 7.4, GSH = 0 mM environment, the micelle structure remained stable in general, and the total release was 15.23% at 48 h. At pH = 5.5, GSH = 0 mM, and pH = 7.4, GSH = 5 mM environment, the total release was 50.2% and 57.85%.	Release of the therapeutic agent occurs in weakly acidic conditions, supporting cancer therapy.	[59]
metal coordination bonds	film	pH 6.0, 7.4	The cumulative release percentage of DFCR from DNMIL is 71.58% at pH 6.0 and 51.08% at pH 7.4. The cumulative release percentage of DFCR from DBMIL is 40.05% at pH 7.4 and 75.48% at pH 6.0.	DBMIL/CS holds great promise in controlled DFCR release in tumor treatment.	[62]
MIL-101(Fe)	bilayer film	pH 5.0, 7.0	The cumulative release of alliin from the NST-NPVA/PLA film after 36 h was 91% at pH 5.0 and 84% at pH 7.0.	The alliin@MIL/NST–NPVA/PLA composite film successfully extended the shelf life of strawberries from 2 days to 7 days	[63]
acylhydrazone bonds and imine bonds	hydrogel	pH 6.5, 7.4	For the hydrogel with PAHy content of 0, 0.2, 0.4, 0.6, 0.8 wt%, the hydrogel could retain 20.3%, 40.06%, 46.21%, 59.48%, and 77.28%, respectively, at pH 7.4. On the other hand, the residual mass rates of hydrogels were 20.16%, 26.52%, 33.92%, 36.28% and 40.35%, respectively, at pH 6.5.	The pH-responsive hydrogel can serve as a controllable and sustained-release carrier for protein drugs.	[70]
boronate ester bond	boronated cyclodextrin framework (BCF)	pH 5.5, 7.4	7.63 ± 0.22% of RGD-EVs were released during 12 h at pH 7.4, and 15.83 ± 0.22% were released at pH 5.5 during the same period.	BCF was able to capture and protect RGD-mEVs, which showed extended release profiles and responsiveness.	[71]
boronate ester bond	polymer dot-coated surface	pH 6.0, 6.8, 7.4	The resistance decreased from 211 ± 9.7 kΩ at pH 7.4 to 73.9 ± 9.4 kΩ and 61.5 ± 11.5 kΩ at pH 6.8 and 6.0.	The Plu-PD coated surfaces can be seamlessly integrated with wireless systems, allowing rapid and accurate cancer diagnosis using smartphones.	[73]
boronate ester bond	nanogel	pH 6.0, 7.4	In PBS buffer at pH 7.4, BAI showed minimal release from BAI@ASPOBA over 48 h. At pH 6.0, the cumulative release of BAI reached 42.4% in the same period.	The constructed BAI@ASPOBA nanogels enhanced the anti-tumor efficacy of BAI, facilitating tumor immunotherapy.	[74]
boronate ester bond and dynamic Schiff	hydrogel	at 37 °C and pH 7.4	Both CS-FPBA-P-gel and CS-FPBA-DBA-P-gel exhibited sustained-release effects in the initial stage, with the cumulative release almost plateauing after 7 h, releasing 72.0 ± 1.9% and 86.1 ± 1.6% of the PAP.	The newly developed hydrogel has ideal antibacterial activity against *Staphylococcus aureus* and *Escherichia coli*, demonstrating its great potential in treating wounds.	[75]

### 3.2. pH-Sensitive Nanoparticles

#### 3.2.1. Metal–Organic Frameworks

Metal–organic frameworks (MOFs) are highly porous materials composed of metal ions or clusters coordinated with organic linkers. They have garnered significant research interest [77]. Due to their pH-sensitive nature in the tumor microenvironment, they are considered ideal candidates for drug delivery applications. The pH response mechanism of MOF is mainly due to the protonation of functional groups within organic ligands or acid-labile coordination bonds. This process may result in structural degradation or increased permeability of the framework, thereby facilitating controlled release of the encapsulated therapeutic agents. For example, titanium-based MOFs (Ti-MOFs) such as MIL-125 and MIL-125-NH_2_, present accelerated 5-fluorouracil release under acidic conditions [78]. Similarly, iron-based MOFs, especially MIL-101 (Fe), have been used in food packaging to achieve pH-responsive alliin release and enhance antibacterial properties [63].

Metal–organic frameworks (MOFs) exhibit diverse compositions, each imparting distinct advantages and limitations for pH-responsive drug delivery (Table 2). In addition to conventional delivery systems, MOFs have been utilized across various biomedical and food applications. Researchers have developed an advanced MOF platform with multiple stimulus responses. These platforms combine enzyme sensitivity or thermal sensitivity with pH responsiveness to achieve more precise controlled release [79,80]. For instance, sodium lignosulfonate-conjugated UiO-66 MOFs enable controlled pesticide release under both acidic and enzymatic conditions, demonstrating the versatility of MOFs in agricultural fields [81].

Overall, MOFs represent a highly promising platform for pH-sensitive delivery, allowing for controlled and site-specific release. Their high surface area, tunable porosity, and ease of functionalization make them particularly valuable in the field of nanomedicine. Although challenges such as complex synthesis and limited long-term stability persist, current research aims to overcome these issues. These efforts expand the potential applications of MOFs in controlled release systems.

#### 3.2.2. Covalent Organic Frameworks

The covalent organic frameworks (COFs) are crystalline porous materials with highly tunable structures and chemical properties. The large surface area and well-defined pore structures of the COFs make them suitable for drug delivery and food safety applications. The key mechanism of pH responsiveness in COFs is the breaking of the acid sensitive bond or the protonation or deprotonation of ionizable groups. This causes structural degradation and controlled release of encapsulated molecules. Recent studies have explored COF-based nanocarriers for targeted drug release. For example, the carboxymethyl starch-gelatin-coated COF has been developed for colon cancer therapy, where the drug release was significantly enhanced at pH 7.4, ensuring targeted release in the intestines [83]. Similarly, another study reported a hydrazone-functionalized COF, which demonstrated efficient doxorubicin (DOX) loading and enhanced anticancer activity due to its pH-triggered release at pH 5.2 [84]. In the field of food safety, COF-based sensors have been designed for detecting contaminants such as foodborne pathogens and heavy metals [89]. Despite these advancements, the challenges remain in the stability of COFs in highly acidic environments because of the rapid degradation of imine-linked COFs. Therefore, future research is advised to develop more stable linkages, such as hydrazone or boronate ester bonds, to further enhance the acid resistance of COFs while maintaining their responsive properties.

#### 3.2.3. Mesoporous Silica Nanoparticles (MSNs)

Mesoporous silica nanoparticles (MSNs) have been extensively utilized in drug delivery and food packaging owing to their tunable pore structure, high biocompatibility, and ease of surface functionalization [90,91]. A key functional advancement is the development of pH-responsive MSNs, which achieve targeted cargo release through modifications that respond to environmental pH. For instance, polymer-capped systems such as Eudragit^®^-S100-coated MSNs effectively shield compounds such as catechin under gastric acid (pH 1.9) but allow site-specific release (up to 90%) at colonic pH (7.4) [92]. Similarly, thiol-functionalized MSNs exhibit accelerated release profiles under mildly acidic conditions (e.g., 81% release at pH 5.5 vs. 55% at pH 6.5 over 48 h), significantly enhancing photostability and efficacy in agrochemical applications [93]. In biomedical contexts, pH-responsive MSNs enable precise antitumor therapy. The nanocarrier DOX@MSN-PEI-AA (DMPA), functionalized with anisamide and polyethyleneimine, facilitates receptor-mediated endocytosis and subsequent protonation-triggered doxorubicin release within acidic tumor microenvironments, demonstrating high efficacy and safety [94]. Dual-stimulus systems further refine release control; for example, MSNs@CMCS-HA, responsive to both pH and hyaluronidase, achieve 63.73% quercetin release at pH 5.0 in the presence of enzyme [95]. In food preservation, MSN-based composites such as CMCS/PVA@MSNs-ε-PL films exhibit antibacterial activity through pH-dependent release of ε-polylysine, effectively disrupting bacterial membranes and showing promise in mitigating *Pseudomonas azotoformans* MN10 contamination [86].

Despite these advantages, unmodified MSNs are prone to premature leakage under harsh conditions, necessitating further surface engineering, such as polymer capping (e.g., chitosan, Eudragit^®^) or hybrid composites to enhance stability, refine release kinetics, and improve biocompatibility. Overall, the integration of pH-responsive designs significantly broadens the applicability of MSNs across biomedical, agricultural, and food packaging sectors.

### 3.3. Polymer

#### 3.3.1. PAA

Poly (acrylic acid) (PAA) is widely recognized as a pH-responsive synthetic polymer owing to the presence of carboxyl groups (–COOH) that undergo protonation and deprotonation in response to environmental pH changes. Under acidic conditions, PAA remains in a compact state due to protonation. At higher pH, the carboxyl groups in PAA dissociate into –COO^−^, causing electrostatic repulsion that leads to polymer swelling, increased water absorption, and the release of encapsulated substances. This swelling behavior makes PAA a useful material for controlled release, such as in drug delivery and food packaging.

In the food field, for example, a pH-responsive film made from PVA and PAA with aminoethyl-phloretin (AEP) added was created to enhance food preservation. The composite film showed strong mechanical and barrier properties, and controlled release of AEP. When applied in pork packaging, it extended shelf life by four days at 25 °C through pH-triggered antimicrobial activity [96]. Similarly, a smart composite coating was designed by grafting polyacrylic acid (PAA) and a bacteriophage-derived endolysin onto ZnO nanocolumns for food-contact surfaces. In this system, the super-hydrophilic PAA layer inhibited initial bacterial adhesion. The coating self-regulated in response to bacterial adhesion; upon substantial accumulation of bacteria, the PAA chains disintegrated to expose the underlying endolysin, which then effectively lysed the compromised pathogens [97]. Another innovative application in the biomedical field involves electrospun PAA-PVA nanofibers for wound dressings. These nanofibers were loaded with bromothymol blue (BTB), a pH-sensitive dye that changes color to green and blue at pH 7 and 8.5, serving as a visual infection indicator. Concurrently, ciprofloxacin was released in response to elevated pH, providing on-demand antibacterial treatment [98].

#### 3.3.2. L100

Eudragit^®^ L100 is widely utilized in the pharmaceutical and food industries owing to its pH-sensitive properties. In pharmaceutical applications, L100 is particularly valuable for controlled drug delivery. It remains insoluble in acidic environments (e.g., the stomach) but dissolves at pH values above 6.0, making it an ideal polymer for colon-targeted oral formulations [14,99]. The research suggested that coating tablets with L100 facilitates targeted drug release [100,101]. This minimizes premature drug dissolution in the upper gastrointestinal tract and enhances localized release in the intestines [102]. Besides oral delivery, L100-based nanoparticles can also enhance the penetration of active drugs into diseased tissues of the skin, highlighting their potential in dermatological applications [103].

In the field of food, L100 has been applied in the intelligent packaging systems to regulate the release of active compounds in response to the changes in environmental pH. Recently, Huang et al. developed a pH-triggered bilayer film consisting of Eudragit^®^ L100, carboxymethyl cellulose, and anthocyanins, which exhibited distinct color changes at pH 6.0, which can achieve real-time monitoring of pork freshness [99]. Moreover, the L100-based coatings have also been used to control the release of antimicrobial agents, such as cinnamon essential oil. These coatings enable faster release at higher pH levels, thereby extending the shelf-life of pork [14].

Though the L100 has been widely used in the field of biomedical and food packaging, the mechanical properties of L100 may require further improvement for some special applications. To address this, composite polymers have been explored by adding other functional polymers, such as polyurethane or chitosan, to enhance their performance [100,104]. The optimization of functional characteristics of L100 is suggested in order to broaden its application in advanced drug delivery and food preservation.

#### 3.3.3. Lignin

The lignin is a biodegradable natural polyphenolic compound composed mainly of p-hydroxyphenyl (H), guaiacyl (G), and syringyl (S) units linked by ether and carbon-carbon bonds. Its oxygen-containing functional groups, including methoxy, hydroxyl, carboxyl, and carbonyl groups, provide high reactivity and pH sensitivity. These properties make it suitable for controlled-release applications. Lignin also exhibits excellent antioxidant and antimicrobial properties [105,106,107]. Recent studies have modified its functional groups to develop lignin-based nanomaterials with enhanced pH-responsive characteristics for drug delivery and antimicrobial applications.

The pH-responsive mechanism in modified lignin is often achieved through integrating acid-sensitive groups to alter the properties of the polymer in response to pH changes. Yi et al. introduced the imidazole via the Mannich reaction and acetylation, creating a system called Ace-SKL-HIS. This achieves the charge reversal in acid to enable the targeted drug release [108]. Gao et al. developed the nanoparticles through conjugating curcumin via an esterification reaction. The ester hydrolysis of the nanoparticles in mildly acidic conditions triggers release [109]. The hydrophilicity and swelling behavior can be controlled by adjusting the hydroxyl content [110].

This pH-sensitive behavior leads to effective applications. Lignin-hollow-nanospheres released only 18% of ibuprofen at gastric pH (1.2) but over 90% at intestinal pH (7.5) within 2–6 h [111]. Ace-SKL-HIS@CUR nanospheres released 76.82% of curcumin at pH 5.7 versus 12.92% at pH 7.4 over 120 h, showing potential for antitumor therapy [108]. Lig-Cur nanoparticles also scavenged ROS and reduced inflammation, indicating utility against oxidative stress [109]. In antimicrobial applications, a lignin-EGCG hydrogel achieved over 96% bactericidal efficacy against *E. coli* and *S. aureus* via synergistic pH-responsive release [112].

In summary, functional group modification enables lignin-based platforms to achieve precise pH-responsive control for advanced drug delivery and antimicrobial applications.

### 3.4. Natural Material

#### 3.4.1. Shellac

Shellac, a natural and biodegradable resin derived from the secretions of the insect *Kerria lacca,* is composed mainly of polyhydroxy polycarboxylic esters, lactones, and anhydrides, with aleuritic and terpenic acids as its major constituents [113]. Owing to its insolubility in acidic to neutral aqueous media and dissolution at elevated pH, shellac serves as an effective enteric coating material suitable for delivery targeted to the intestinal [114].

This property has facilitated its widespread use in delivery systems. For example, hybrid systems such as double-network gels, developed for co-encapsulating probiotics and capsaicin, form dense microstructures through hydrophobic interactions and disulfide bonds during gelation. A specific formulation with 1.5% GDL and 4% shellac was shown to exhibit the densest architecture and maintain high structural integrity during in vitro digestion, thereby enhancing functional performance [115]. Similarly, shellac-zein nanoparticles have been demonstrated to enable efficient intestinal release of curcumin while protecting it from degradation in gastric conditions [116].

Recent efforts have focused on combining shellac with polysaccharides or proteins and employing chemical cross-linking to enhance its mechanical properties, thereby improving both stability and functional performance [117].

#### 3.4.2. Alginate

Alginate, a naturally occurring anionic polysaccharide derived from brown seaweed, exhibits distinct pH-responsive behavior due to the protonation and deprotonation of its carboxyl groups. It remains insoluble and contracts under acidic conditions but swells and dissolves in alkaline environments, making it highly suitable for controlled-release applications [29]. This property has been developed for the targeted delivery of proteins, active ingredients [118,119], and probiotics, protecting them from acidic degradation [120].

Ionotropically gelled alginate hydrogels, typically crosslinked with calcium ions, are widely used in oral delivery systems to improve encapsulation efficiency and enable pH-dependent intestinal release. For instance, sodium alginate and whey protein composite beads enhance the stability and controlled release of theaflavins, providing effective gastric protection and promoting intestinal delivery [121]. Similarly, alginate–protein composites facilitate the mucoadhesive release of hydrophobic nutrients such as limonin, significantly improving their bioaccessibility [122]. A recent study developed a coaxial 3D-printed probiotic delivery system using a starch core to encapsulate *Bifidobacterium bifidum* and an alginate/pectin shell, which provided high acid protection (83.1% viability in gastric fluid compared to complete loss of free cells) and enabled targeted release in the intestine, demonstrating great potential for functional foods [123].

However, challenges such as rapid dissolution under alkaline conditions and inherently weak mechanical strength can compromise sustained release performance. To address these issues, alginate has been blended with polymers, including chitosan, κ-carrageenan, or gelatin, to reinforce the network structure [124]. Future studies should focus on optimizing such hybrid formulations and modification strategies to enhance mechanical robustness and achieve precise release profiles for broader applications.

#### 3.4.3. Chitosan

Chitosan, a cationic polysaccharide derived from the deacetylation of chitin, exhibits pH-responsive behavior due to the protonation and deprotonation of its amino groups (–NH_2_). This enables dissolution under acidic conditions (pH < 6.5) and precipitation in neutral/alkaline environments, making it highly valuable for controlled-release applications across multiple fields [53,125]. Moreover, chitosan has been widely used due to its function in reducing the premature release of biological activity in nanocomposite systems and enhancing antibacterial properties [126,127].

In food packaging, chitosan-based materials enable spoilage-triggered release of antimicrobial agents. For example, chitosan-fucoidan films crosslinked via Schiff-base bonds accelerated cinnamaldehyde release at pH 5, enhancing antibacterial activity by 2.3-fold compared to pH seven and extending litchi shelf life by 8 days [53]. Similarly, chitosan/polyaspartic acid nanofibers achieved 56% cumulative release of tea polyphenols at pH 5.0, effectively inhibiting *Botrytis cinerea* on strawberries [128]. Besides food applications, chitosan’s pH sensitivity has also been applied in agriculture for nutrient delivery. Specifically, a genipin-crosslinked chitosan hydrogel (GE-CSG@Fe) served as a carrier for intelligent iron fertilizer release, providing slow release under neutral conditions and rapid release in acidic environments. This system enhanced crop growth and improved nutrient absorption [129]. In pharmaceutical applications, Chitosan/PVA/GO hydrogels swelled at pH 4.0 and released over 80% of cefixime within 8.5 h in simulated gastric, highlighting their potential for pH-responsive drug delivery [130]. Pourmadadi et al. integrated MoS_2_ into chitosan nanocomposites, increasing drug loading and encapsulation efficiency [131].

#### 3.4.4. Oxidized Starch

Oxidized starch modified by introducing carboxyl and carbonyl groups exhibits distinct pH-responsive behavior [132]. This behavior results from the protonation and deprotonation of its ionizable functional groups [133,134]. In the delivery of active ingredients, for example, TEMPO-oxidized starch demonstrated pH-triggered swelling, with swelling ratios increasing from 15.6 at pH 2 to 31.6 at pH 7, highlighting their potential for nutrient delivery [134]. Another example is oxidized starch microgels (OSM), which effectively protected quercetin-loaded nanoparticles under gastric conditions and facilitated their release in the intestine [133].

The pH sensitivity of oxidized starch has widespread applications in delivery systems. However, oxidized starch is often limited by its mechanical fragility and susceptibility to hydrolysis under extreme pH conditions [135]. To overcome these limitations, strategies such as blending with chitosan or crosslinking with metal ions (e.g., Zn^2+^) have been employed to enhance stability and modulate release kinetics [136,137]. Furthermore, both the degree of oxidation and the botanical source strongly affect its performance. Optimization could balance pH responsiveness and structural integrity [138].

#### 3.4.5. Oxidized Cellulose

Oxidized cellulose (OC) demonstrates pH-responsive behavior due to the protonation and deprotonation of its functional groups. This mechanism allows solubility and swelling to be adjusted under different pH conditions, which makes it suitable for controlled-release applications. The oxidation degree dictates performance by improving key properties such as water solubility, negative charge, and gel-forming ability [139].

OC-based delivery systems have been widely applied for intestinal targeted release. They maintain stability in the acidic conditions and rapidly swell under intestinal conditions, allowing for targeted delivery. Xie et al. developed the TEMPO-oxidized cellulose beads (OCBs), which effectively retained drugs at pH 1.2 and released them at pH 7.0, following zero-order release kinetics [140]. In the fields of agricultural applications, a hybrid hydrogel composed of TEMPO-oxidized cellulose nanofibers (CNFs) and MIL-100(Fe) MOF exhibited dual temperature/pH responsiveness, enabling intelligent release of urea. At an optimal formulation, it demonstrated high swelling capacity (37 g/g), water retention (22.78%), and sustained release (40.84%), significantly improving wheat growth and photosynthetic parameters [141]. In functional food applications, a biomimetic cell wall system constructed from TEMPO-oxidized cellulose effectively protected fucoxanthin (FX) in gastric acid and triggered its targeted release in the intestine, markedly enhancing stability and bioavailability [142].

Future efforts should prioritize the development of multifunctional OC composites that integrate pH sensitivity, mechanical robustness, and environmental adaptability to broaden their utility in biomedical, agricultural, and food applications.

## 4. Release Kinetic Models

The analysis of release kinetics is fundamental to the optimization of delivery systems, as it elucidates key release mechanisms such as diffusion, swelling, polymer degradation, and structural transformations [143,144]. To quantitatively analyze these processes, various kinetic models are commonly employed, including zero-order, first-order, Higuchi, Korsmeyer–Peppas, and Peppas–Sahlin equations. Among them, the Korsmeyer–Peppas model is applicable to the drug release of polymer carriers controlled by diffusion and polymer chain relaxation [145,146]. In the case of polymer erosion-controlled release, the Peppas–Sahlin model offers a more precise explanation of the underlying mechanism [147].

For pH-responsive carriers, release behavior often follows Fickian, non-Fickian, or anomalous transport kinetics, reflecting the interaction between diffusive and polymer relaxation processes. The release models and corresponding parameters for different carriers are summarized in Table 3. A study on alginate-based hydrogels and Eudragit^®^-coated nanoparticles demonstrated that external pH changes significantly affected polymer swelling or dissolution, thereby modulating the rate of drug diffusion [92,146]. For example, imatinib-loaded Fe_3_O_4_@SiO_2_ nanoparticles displayed accelerated release at pH 5.6 compared to pH 7.4. This behavior can be explained by the Peppas–Sahlin model, as shown in Table 3 [147]. Similarly, Korsmeyer–Peppas kinetics effectively described the curcumin release from chitosan-based systems under pH-sensitive conditions, indicating a diffusion-dominated release mechanism [148].

In numerous pH-sensitive films and hydrogel systems, first-order kinetics are frequently observed, as release rates are often proportional to the remaining drug concentration within the matrix. For instance, ampicillin-loaded films composed of alginate, hyaluronic acid, and gelatin exhibited higher release rates at pH 1.2 than at pH 7.4, with the release profile fitting a first-order kinetic model with a high correlation coefficient, as illustrated in Table 3 (R^2^ = 0.966) [146]. Consistent with this, sodium alginate-methacrylic acid hydrogels developed for ulcerative colitis also exhibited first-order release behavior, governed primarily by pH-dependent swelling and diffusion [149].

The selection of the most appropriate kinetic model depends on several factors, including carrier composition, the specific nature of pH-triggered polymer responses, and the dominant release mechanism. While the Korsmeyer–Peppas and Peppas–Sahlin models are ideal for systems where polymer relaxation and erosion play major roles, the first-order and Higuchi models are better suited for systems where dissolution and diffusion are the primary drivers of release. Together, these models not only enable accurate prediction of drug release profiles but also support the rational design of advanced pH-responsive systems for targeted and sustained therapeutic applications.

## 5. Application

### 5.1. Application of pH-Responsive Systems in Nutrition Release

pH-responsive delivery systems allow precise control over vitamin release by exploiting physiological pH variations to induce ionization, swelling, or degradation of the carrier matrix. For water-soluble vitamins, systems functionalized with ionizable groups demonstrate suppressed release under acidic conditions and enhanced release in the intestine. Carboxylated cellulose microspheres (CCMs) minimized vitamin B_12_ release at gastric pH (1.2) while promoting its release at intestinal pH (7.4), governed by the ionization equilibrium of carboxyl groups [150]. Similarly, gelatin-g-poly(acrylic acid)/LDH hydrogels released 31% of vitamin B_12_ at pH 7.4 compared to 24% at pH 1.2, attributable to polymer matrix expansion under alkaline conditions [151]. Algal protein/Ca^2+^ hydrogels released 83% of vitamin B_6_ at pH 7.4, with release kinetics following either Fickian diffusion or anomalous transport per the Peppas–Sahlin model, while acidic conditions (pH 2.1) limited release due to network contraction [152].

For fat-soluble vitamins, pH-responsive encapsulation enhances stability and enables targeted intestinal delivery. A gelatin-pectin (GA-HMP/LMP) complex coacervate remained stable in gastric acid, inhibiting premature release, and facilitated sustained intestinal release via electrostatically modulated structural changes [153]. Arachin-basil seed gum gels achieved 91.7% encapsulation efficiency for vitamin D_3_, with controlled release during simulated gastric digestion and a bioaccessibility of 32.9% [154].

Furthermore, pH-responsive systems allow co-delivery of vitamins and probiotics. A self-assembled hydrogel composed of gellan gum, κ-carrageenan, and chitosan significantly suppressed the release of both spores (28.42%) and folic acid (45.14%) under acidic conditions, while enabling sustained release in neutral and alkaline intestinal environments [155]. Similarly, a soy protein isolate (SPI)-arabinoxylan hydrogel minimized riboflavin leakage in the stomach due to its dense network, yet achieved 98.04% release in the intestine through pH-triggered swelling [156]. These systems utilize pH-dependent structural changes to synchronize the delivery of nutrients and probiotics, thereby improving bioavailability and functional efficacy.

In summary, pH-responsive carriers represent a robust strategy for overcoming bioavailability limitations and achieving site-specific nutrient release through controlled mechanisms. Although biopolymers (such as gelatin and pectin) are food-grade, their mechanical strength and processing stability are challenges for large-scale production. Large-scale preparation of hydrogels is expensive, and it must be ensured that they do not leak prematurely during food processing.

### 5.2. Application of pH-Responsive Systems in Flavor Encapsulation and Release

The pH-responsive flavor-controlled release system utilizes the property changes in encapsulation materials at different pH levels for intelligent management. This system works by retaining aromas through its structure during storage, while triggering targeted release in specific acidic environments to enhance the flavor profile [157,158].

By combining physical encapsulation and chemical cross-linking strategies, Yu et al. successfully developed chitosan-based pH-responsive hollow microspheres (CHMP), providing an innovative solution for the stable storage of flavor substances. Under neutral conditions, the stable Schiff-base bonds and encapsulated structure effectively retain cinnamaldehyde, enabling long-term storage. Results demonstrated that after 48 days of open storage at room temperature, the retention rate of cinnamaldehyde remained as high as 85% [158]. Similarly, Xu et al. developed a metal polyphenol-based O/W emulsion to form tunable core–shell microcapsules with high central loading capacity. It exhibits a distinct pH response behavior, releasing slowly under neutral conditions and rapidly under acidic conditions [157]. Furthermore, pH-responsive chitosan microspheres loaded with aminopeptidase (A-CM) were designed for precise enzyme release during cheese ripening. These microspheres retained the enzyme at higher pH values (5.5–6.5) during processing and triggered its release at reduced pH (5.0–5.5) in the ripening phase, likely due to pH-dependent electrostatic interactions. The addition of A-CM to cheese resulted in the highest proteolytic activity, a significant increase in key flavor compounds, and superior sensory quality, demonstrating its considerable potential for flavor modulation [159].

In summary, materials such as chitosan have advantages, but the industrial production cost of microcapsules is too high. Its applications may be limited to high-value-added products such as cheese.

### 5.3. Application of pH-Responsive Systems in Food Preservation

pH-responsive systems represent a promising strategy for intelligent food preservation, leveraging spoilage-induced pH changes to achieve targeted release of antimicrobial and antioxidant agents. These systems enhance preservation efficiency by responding specifically to metabolites produced during microbial growth or oxidative reactions.

**Figure 3:**
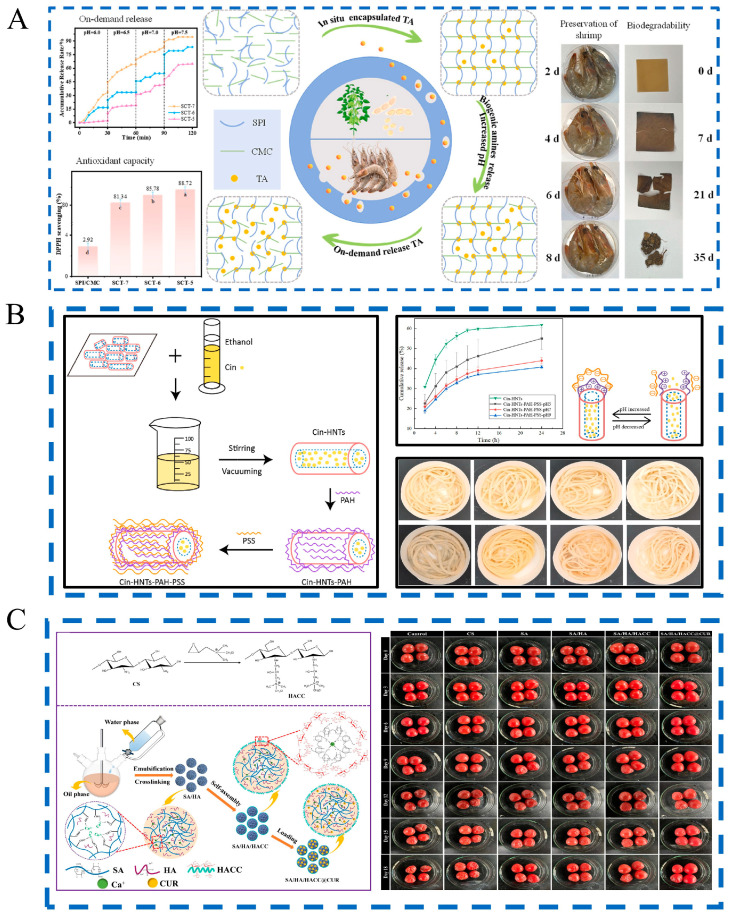
The application of pH-responsive release technology in food preservation. (**A**) pH-triggered on-demand tannic acid release for shrimp preservation [164], lowercase letters indicate significant differences, (**B**) pH-sensitive nanocomposites for fresh noodle preservation [163], (**C**) pH-sensitive microspheres for fruit preservation [161].

pH-responsive release systems are strategically designed to activate under specific acidity levels prevalent in different food spoilage scenarios. Acid-triggered systems are particularly effective in acidic food environments. For instance, a carboxymethyl cellulose film incorporated with chlorine dioxide exhibited 43% release at pH 4.0, significantly inhibiting *Aspergillus niger* and reducing strawberry decay incidence from 100% to 20% [160]. Similarly, sodium alginate/hydroxyapatite/quaternary ammonium chitosan microspheres encapsulated with curcumin demonstrated accelerated release under acidic conditions, providing sustained antibacterial and antioxidant protection for cherry tomatoes (Figure 3C) [161]. Furthermore, the structural collapse of ZIF-8 under acidic conditions enabled tea polyphenol-loaded nanofibers to achieve 73.41% release at pH 5.0, markedly extending the shelf life of both strawberries and salmon [162]. Notably, polyelectrolyte-capped halloysite nanotubes loaded with cinnamaldehyde also exhibited clear pH-dependent release behavior, effectively inhibiting microbial growth in fresh wheat noodles (Figure 3B) [163].

In contrast, for foods that undergo spoilage characterized by neutral to alkaline pH shifts, alternative release systems have been developed. A composite film consisting of soy protein isolate, carboxymethyl cellulose, and tannic acid responded to biogenic amines and significantly extended the shelf life of Pacific white shrimp by 8 days, leveraging its enhanced antioxidant capacity (as indicated by the different letters in Figure 3A [164]. Similarly, a chitosan/gelatin-based film incorporating carvacrol-loaded halloysite nanotubes displayed rapid release under alkaline conditions, effectively inhibiting *Escherichia coli* and *Staphylococcus aureus* in pork [165]. Additionally, coaxial electrospun films containing cinnamon essential oil showed high release (98.2%) at pH 6.25, extending the shelf life of griskin by three days [14]. These advanced systems demonstrate pH response behaviors, aiming to enhance preservation effectiveness.

Smart packaging films from CMC and chitosan are highly feasible owing to their low cost and easy processing. Key challenges include uniformly dispersing nanofillers and ensuring the final product meets industrial mechanical standards and migration safety regulations.

## 6. Safety and Regulation Rules

The safe application of pH-responsive controlled-release technology in food necessitates rigorous safety assurance, encompassing material toxicology, migration risks, and regulatory compliance. Material safety is demonstrated through various approaches, including the use of pre-approved food-grade polymers. For instance, chitosan (CS) and sodium alginate (SA) are widely recognized as non-toxic, biodegradable, and antibacterial biopolymers, making them highly suitable for food packaging applications [3,128]. Similarly, shellac, a natural resin, has been extensively studied and used for probiotic encapsulation to enhance their survival in the gastrointestinal tract due to its insolubility in gastric acid but solubility at intestinal neutral pH [115]. For novel or modified materials, systematic toxicological assessment is essential to ensure safety. A representative example includes TEMPO-oxidized cellulose nanofiber (TOCNF)-based hydrogel films, in which the residual acrylamide monomer was controlled below 0.02% during synthesis, significantly lower than the FDA regulatory limit of 0.05%. HPLC analysis further confirmed no detectable migration of the monomer into packaged mango samples, supporting its safety for food packaging applications [166]. Biocompatibility tests established in the biomedical field, such as cytotoxicity assays, have been adopted to evaluate the safety of materials for food-related applications. For instance, lignin-loaded PVA-CS hydrogels exhibited excellent biocompatibility, with extract-treated cells showing survival rates exceeding 100% and enhanced proliferation, consistent with ISO 10993-12-2021 standards [167]. Similarly, PAA/SA/CMC/PS/CUR composite hydrogels and glass fiber-reinforced scaffolds demonstrated significant cytocompatibility and promoted cell growth, indicating preliminary biosafety for potential use in food-controlled release systems [168]. Key risks include unintended release of actives due to system instability. Any new or modified materials used in direct food contact must meet the regulatory safety standards established by authorities such as the FDA (United States) or EFSA (European Union). Specifically, materials must adhere to defined safety evaluation standards (e.g., 21 CFR 177.2510), which include requirements related to temperature, chemical exposure, and contact duration [19].

## 7. Conclusions and Future Perspectives

pH-responsive materials represent a promising class of smart delivery systems capable of releasing active ingredients in response to microenvironmental pH variations. These systems operate through mechanisms such as protonation/deprotonation and the cleavage of acid-labile covalent bonds–including imines, disulfide bonds, acyl hydrazones, borate esters, and metal-ligand coordination bonds. Commonly employed carriers encompass metal–organic frameworks (MOFs), covalent organic frameworks (COFs), mesoporous silica nanoparticles (MSNs), and functional polymers bearing carboxyl or amino groups, such as polyacrylic acid, chitosan, shellac, and L100. These materials have demonstrated effective application in nutrient encapsulation, flavor protection, and active food packaging.

However, the path to industrial implementation is hampered by several challenges. These include the insufficient mechanical strength of natural polymers, scalability and safety concerns of synthetic nanocarriers, and the unreliable release kinetics in complex real food matrices. To address these issues, future material-focused research should prioritize enhancing controllability, scalability, and storage stability: (1) tailoring dynamic covalent bonds for precise release control; (2) constructing multi-component composites for enhanced stability; (3) employing core–shell nanostructures to prevent leakage; and (4) optimizing green crosslinking strategies for durability.

Looking beyond incremental material improvements, the integration of artificial intelligence (AI) and biomimetic design is poised to enable a paradigm shift towards truly intelligent packaging. AI’s pivotal role lies in addressing the core challenges of predictability and control through three key avenues: (1) precise molecular design: machine learning can analyze vast datasets linking polymer structures to performance, accelerating the discovery of next-generation pH-responsive materials. (2) multiscale modeling of release kinetics: AI can establish accurate, system-level simulations to predict release behavior under complex, real-world conditions (e.g., fluctuating temperature and pH), overcoming the limitations of traditional physical models. (3) adaptive and personalized release control: By deploying pre-trained AI models, packaging can dynamically assess food status via real-time sensor data (e.g., pH, temperature), intelligently adjusting trigger thresholds and release rates to achieve dynamic responsiveness.

For any of these innovative materials to transition into real-world applications, especially those intended for direct food contact, compliance with stringent regulatory safety standards (e.g., from the FDA or EFSA) is imperative. Therefore, establishing robust safety evaluation protocols and clear regulatory guidelines will be essential to ensure standardized assessment and scaled production, thereby accelerating the safe and sustainable adoption of pH-responsive technologies in advanced food systems.

## Figures and Tables

**Table 2:** Benefits and limitations of acid-sensitive nanoparticles.

Material	Advantage	Disadvantage	Application	Reference
UiO-66	Rapid and targeted release	—	Pesticide targeting release	[81]
MIL-101(Fe)	biocompatibility and high porosity	—	Food packaging	[63]
MIL-125	Superior biocompatibility	Complex synthesis	Cancer therapy	[78]
Co-MOF	Enhanced UV-visible barrier, anti-migration performance of pigment, and thermal stability.	—	Monitor freshness	[82]
COF-5Fu	High loading rate	Unstable in harsh acidic conditions	Colon cancer therapy	[83]
Hydrazone-decorated NCOFs	Excellent and intelligent sustained-release effect	Introduces some toxic substances	Cancer therapy	[84]
Spinosad@MSNs-PLA	High loading capacity (38.6%improved photostability of spinosad) and improved photostability of spinosad	—	Biopesticides release	[85]
CMCS/PVA@MSNs-ε-PL	Improved the dispersion stability	—	Food preservation	[86]
Functionalized Mesoporous Silica Nanoparticles	High sensitivity, specificity,	Need modification	AFB1 detection in food	[87]
MSN	Target-induced release	—	Ofloxacin detection	[88]

**Table 3:** Release models and parameters for different carriers.

Materials	Models	Parameters	pH	R^2^	*n*	Main Mechanism	Reference
Alginate/hyaluronic acid/gelatin ternary	first order	k_1_	7.4	0.966	—	drug concentration	[146]
	Korsmeyer-peppas	K_k-p_	7.4	0.935	1.26	swelling and relaxation of ternary-blended polymeric matrix	
			1.2	0.928	1.28		
							
(PVP)_6.25_-CTAB-SiOH	Peppas–Sahlin	m = 0.5, k_1_, k_2_	6.4	0.995	—	Fickian diffusion and Case II transport	[145]
						
(PVP)_6.25_-(SiOH)_2_	Peppas–Sahlin	m = 0.48, k_1_, k_2_	2.8	0.9944	—	diffusion–controlled processes	
	Ritger–Peppas	AIC	2.8	0.9725	0.27		
	First order	k_1_, AIC	2.8	0.9366	—		
							
MSNPs/CHT	Korsmeyer-Peppas	K_k–p_	1.9, 5.5, 7.4	0.9844	0.2662	Fickian diffusion	[92]
NH2-MSNPs/CHT	Korsmeyer-Peppas			0.9146	0.3073	Fickian diffusion	
MSNPs/CHT@EUS-100	Korsmeyer-Peppas			0.9937	1.886	supercase II transport	
							
Alginate-methacrylic acid hydrogels	First order	k_1_	1.2	0.9962	—	Fickian diffusion	[149]
			7.4	0.9696	—		

## Data Availability

The original contributions presented in the study are included in the article; further inquiries can be directed to the corresponding authors.

## References

[1] Karim N., Shishir M.R.I., Marappan G., Khan S., Hashim S.B.H., Aalim H., Arslan M., Tahir H.E., Li Z., Zhai X. (2024). Recent advances in delivering mangosteen-based phytochemicals using promising micro/nanocarriers: Formulation, outcomes, and perspectives. Trends Food Sci. Technol..

[2] Lu X., Wang C., Zhao M., Wu J., Niu Z., Zhang X., Simal-Gandara J., Suntar I., Jafari S.M., Qiao X. (2022). Improving the bioavailability and bioactivity of garlic bioactive compounds via nanotechnology. Crit. Rev. Food Sci. Nutr..

[3] Guo J., Qiu Y., Zhang J., Xue C., Zhu J. (2025). A review on polysaccharide-based delivery systems for edible bioactives: pH responsive, controlled release, and emerging applications. Int. J. Biol. Macromol..

[4] Lin L., Mei C., Shi C., Li C., Abdel-Samie M.A., Cui H. (2023). Preparation and characterization of gelatin active packaging film loaded with eugenol nanoparticles and its application in chicken preservation. Food Biosci..

[5] Surendhiran D., Li C., Cui H., Lin L. (2021). Marine algae as efficacious bioresources housing antimicrobial compounds for preserving foods—A review. Int. J. Food Microbiol..

[6] Herrera-Balandrano D.D., Chai Z., Beta T., Feng J., Huang W. (2021). Blueberry anthocyanins: An updated review on approaches to enhancing their bioavailability. Trends Food Sci. Technol..

[7] Sobhy M., Abdelkarim E.A., Hussein M.A., Aziz T., Al-Asmari F., Alabbosh K.F., Cui H., Lin L. (2025). Essential oils as antibacterials against multidrug-resistant foodborne pathogens: Mechanisms, recent advances, and legal considerations. Food Biosci..

[8] Herrera-Balandrano D.D., Wang J., Chai Z., Zhang X., Wang J., Wang N., Huang W. (2023). Impact of in vitro gastrointestinal digestion on rabbiteye blueberry anthocyanins and their absorption efficiency in Caco-2 cells. Food Biosci..

[9] Virk M.S., Virk M.A., Gul M., Awais M., Liang Q., Tufail T., Zhong M., Sun Y., Qayum A., El-Salam E.A. (2025). Layer-by-layer concurrent encapsulation of probiotics and bioactive compounds with supplementation in intermediary layers: An establishing instrument for microbiome recharge, core safety, and targeted delivery. Food Hydrocoll..

[10] Zhang W., Liu L., Zhao Y., Liu T., Bai F., Wang J., Xu H., Gao R., Jiang X., Xu X. (2024). Interactions between phosvitin and aldehydes affect the release of flavor from Russian sturgeon caviar. Food Chem..

[11] Cui H., Wang Y., Li C., Chen X., Lin L. (2021). Antibacterial efficacy of *Satureja montana* L. essential oil encapsulated in methyl-β-cyclodextrin/soy soluble polysaccharide hydrogel and its assessment as meat preservative. LWT.

[12] Li C., Chen W., Siva S., Cui H., Lin L. (2021). Electrospun phospholipid nanofibers encapsulated with cinnamaldehyde/HP-β-CD inclusion complex as a novel food packaging material. Food Packag. Shelf Life.

[13] Zhang J., Huang X., Zhang J., Liu L., Shi J., Muhammad A., Zhai X., Zou X., Xiao J., Li Z. (2022). Development of nanofiber indicator with high sensitivity for pork preservation and freshness monitoring. Food Chem..

[14] Zhang J., Zhang J., Huang X., Shi J., Muhammad A., Zhai X., Xiao J., Li Z., Povey M., Zou X. (2023). Study on cinnamon essential oil release performance based on pH-triggered dynamic mechanism of active packaging for meat preservation. Food Chem..

[15] Li C., Bai M., Chen X., Hu W., Cui H., Lin L. (2022). Controlled release and antibacterial activity of nanofibers loaded with basil essential oil-encapsulated cationic liposomes against Listeria monocytogenes. Food Biosci..

[16] Cui H., Lu J., Li C., Rashed M.M.A., Lin L. (2022). Antibacterial and physical effects of cationic starch nanofibers containing carvacrol@casein nanoparticles against Bacillus cereus in soy products. Int. J. Food Microbiol..

[17] Lin L., Wu J., Li C., Chen X., Cui H. (2022). Fabrication of a dual-response intelligent antibacterial nanofiber and its application in beef preservation. LWT.

[18] Zhang X., Wang Z., Huang X., Hu X., Li Y., Zhou Y., Wang X., Zhang R., Wei X., Zhai X. (2023). H-Bond Modulation Mechanism for Moisture-driven Bacteriostat Evolved from Phytochemical Formulation. Adv. Funct. Mater..

[19] Zhang J., Zhang J., Zhang R., Huang X., Li Z., Zhai X., Shen T., Shi J., Zou X. (2025). Preparation of photodynamic-controlled release packaging for pork preservation and its visualization. Food Chem..

[20] Zhang W., Luo Z., Wang A., Gu X., Lv Z. (2021). Kinetic models applied to quality change and shelf life prediction of kiwifruits. LWT.

[21] Xia J., Zou B., Liu F., Wang P., Yan Y. (2022). Sensitive glucose biosensor based on cyclodextrin modified carbon nanotubes for detecting glucose in honey. J. Food Compos. Anal..

[22] Wei C., Wang Q., Weng W., Adu-Frimpong M., Toreniyazov E., Ji H., Xu X., Yu J. (2022). Enhanced oral bioavailability and anti-hyperuricemic activity of liquiritin via a self-nanoemulsifying drug delivery system. J. Sci. Food Agric..

[23] Wei B., Yin L., Shi K., Zou J., Fan Z., Zhu S., Xu B. (2025). Design of Starch Coatings to Control the In Vitro Release of Insulin From Chitosan Hydrogels. Starch-Stärke.

[24] Yuan J., Zhu Y., Wang J., Liu Z., Zhang T., Li P., Qiu F. (2025). Conversion of agricultural waste biomass resource into high-added-value composite and its potential for boosting synergistic removal of ammonia nitrogen in practical water. Food Bioprod. Process..

[25] Feng J., Liu D., Wang Z., Li C., Huang W., Liu S., Li Y. (2025). Interpenetrating network hydrogels loaded with nanostructured lipid carriers for curcumin delivery: Impact of dual crosslinking with genipin and calcium ions. Food Res. Int..

[26] Su Y., Chen Y., Zhang L., Adhikari B., Xu B., Li J., Zheng T. (2022). Synthesis and characterization of lotus seed protein-based curcumin microcapsules with enhanced solubility, stability, and sustained release. J. Sci. Food Agric..

[27] Hamadou A.H., Zhang J., Chao C., Xu B. (2022). Stability of rutin using pectin-chitosan dual coating nanoliposomes. LWT.

[28] Li C., Liu D., Huang M., Huang W., Li Y., Feng J. (2022). Interfacial engineering strategy to improve the stabilizing effect of curcumin-loaded nanostructured lipid carriers. Food Hydrocoll..

[29] Man Y., Zhou C., Adhikari B., Wang Y., Xu T., Wang B. (2022). High voltage electrohydrodynamic atomization of bovine lactoferrin and its encapsulation behaviors in sodium alginate. J. Food Eng..

[30] Al-Maqtari Q.A., Al-Gheethi A.A.S., Ghaleb A.D.S., Mahdi A.A., Al-Ansi W., Noman A.E., Al-Adeeb A., Odjo A.K.O., Du Y., Wei M. (2022). Fabrication and characterization of chitosan/gelatin films loaded with microcapsules of Pulicaria jaubertii extract. Food Hydrocoll..

[31] Yang Z., Li M., Li Y., Li Z., Huang X., Wang X., Shi J., Zou X., Zhai X., Povey M. (2023). Improving properties of Litsea cubeba oil Pickering emulsion-loaded gelatin-based bio-nanocomposite film via optimizing blending ratio: Application for mango preservation. Food Hydrocoll..

[32] Virk M.S., Virk M.A., Liang Q., Sun Y., Zhong M., Tufail T., Rashid A., Qayum A., Rehman A., Ekumah J.N. (2024). Enhancing storage and gastroprotective viability of Lactiplantibacillus plantarum encapsulated by sodium caseinate-inulin-soy protein isolates composites carried within carboxymethyl cellulose hydrogel. Food Res. Int..

[33] Liu Y., Liang Q., Liu X., Raza H., Ma H., Ren X. (2022). Treatment with ultrasound improves the encapsulation efficiency of resveratrol in zein-gum Arabic complex coacervates. LWT.

[34] Lyu X., Wang X., Wang Q., Ma X., Chen S., Xiao J. (2021). Encapsulation of sea buckthorn (*Hippophae rhamnoides* L.) leaf extract via an electrohydrodynamic method. Food Chem..

[35] Chen C., Chen Z., Zhong Q. (2022). Caseinate nanoparticles co-loaded with quercetin and avenanthramide 2c using a novel two-step pH-driven method: Formation, characterization, and bioavailability. Food Hydrocoll..

[36] Zhang J., Hassane Hamadou A., Chen C., Xu B. (2023). Encapsulation of phenolic compounds within food-grade carriers and delivery systems by pH-driven method: A systematic review. Crit. Rev. Food Sci. Nutr..

[37] Du H., Sun X., Chong X., Yang M., Zhu Z., Wen Y. (2023). A review on smart active packaging systems for food preservation: Applications and future trends. Trends Food Sci. Technol..

[38] Kumar S., Begum S., Baishya H., Das P., Dutta D. (2025). Chitosan-based intelligent freshness indicators for monitoring food quality: A comprehensive review. Int. J. Biol. Macromol..

[39] Wu Z., Xu M., He W., Li X., Qiu C., Zhang J. (2024). Unraveling the Physicochemical Properties and Bacterial Communities in Rabbit Meat during Chilled Storage. Foods.

[40] Fan Y., Schneider K.R., Sarnoski P.J. (2022). Determining spoilage of whiteleg shrimp (Litopanaeus vannemei) during refrigerated storage using colorimetric strips. Food Chem. X.

[41] Guo Y., Ma C., Du L., Xu Y., Yang X. (2025). Research Progress on the Application of Food Colloids in Precise Targeted Delivery of Drugs and Bioactive Compounds. Gels.

[42] Mastromatteo M., Mastromatteo M., Conte A., Del Nobile M.A. (2010). Advances in controlled release devices for food packaging applications. Trends Food Sci. Technol..

[43] Zhu Z.A.-O., Zhou S., Zhou X., Mo S.A.-O., Zhu Y.A.-O., Zhang L., Tang S.A.-O., Fang Z., Fan Y. (2022). Effective Remediation of Arsenic-Contaminated Soils by EK-PRB of Fe/Mn/C-LDH: Performance, Characteristics, and Mechanism. Int. J. Environ. Res. Public Health.

[44] Cheng M., Cui Y., Guo Y., Zhao P., Wang J., Zhang R., Wang X. (2023). Design of carboxymethyl chitosan-reinforced pH-responsive hydrogels for on-demand release of carvacrol and simulation of release kinetics. Food Chem..

[45] Gholizadeh A., Kermani M., Gholami M., Farzadkia M. (2013). Kinetic and isotherm studies of adsorption and biosorption processes in the removal of phenolic compounds from aqueous solutions: Comparative study. J. Environ. Health Sci. Eng..

[46] Siano F.A.-O., Sammarco A.S., Fierro O., Castaldo D., Caruso T.A.-O., Picariello G.A.-O., Vasca E.A.-O. (2023). Insights into the Structure-Capacity of Food Antioxidant Compounds Assessed Using Coulometry. Antioxidants.

[47] Moorthy K., Chang K.C., Wu W.J., Hsu J.Y., Yu P.J., Chiang C.K. (2021). Systematic Evaluation of Antioxidant Efficiency and Antibacterial Mechanism of Bitter Gourd Extract Stabilized Silver Nanoparticles. Nanomaterials.

[48] Pisoschi A.A.-O., Pop A., Cimpeanu C., Predoi G. (2016). Antioxidant Capacity Determination in Plants and Plant-Derived Products: A Review. Oxidative Med. Cell. Longev..

[49] Imohiosen F.A., Ofudje E.A., Al-Ahmary K.M., Al-Mhyawi S.R., Alshdoukhi I.F., Alrahili M.R., Alsaiari A.A., Din S.U. (2024). Pharmaceutical effluent degradation using hydrogen peroxide-supported zerovalent iron nanoparticles catalyst. Sci. Rep..

[50] Cui H., Cheng Q., Li C., Khin M.N., Lin L. (2023). Schiff base cross-linked dialdehyde β-cyclodextrin/gelatin-carrageenan active packaging film for the application of carvacrol on ready-to-eat foods. Food Hydrocoll..

[51] Heras-Mozos R., Gavara R., Hernández-Muñoz P. (2022). Responsive packaging based on imine-chitosan films for extending the shelf-life of refrigerated fresh-cut pineapple. Food Hydrocoll..

[52] Heras-Mozos R., Gavara R., Hernández-Muñoz P. (2021). Development of antifungal biopolymers based on dynamic imines as responsive release systems for the postharvest preservation of blackberry fruit. Food Chem..

[53] Liang F., Liu C., Geng J., Chen N., Lai W., Mo H., Liu K. (2024). Chitosan–fucoidan encapsulating cinnamaldehyde composite coating films: Preparation, pH-responsive release, antibacterial activity and preservation for litchi. Carbohydr. Polym..

[54] Zhou G.-s., Zhang T., Jin Y., Zhang G.-f., Kong B., Chen D.-y., Wang G.-x., Wang Z.-s., Qi M.-y., Zhang D.-x. (2025). A carboxymethyl chitosan /trans-2-hexenal Schiff’s base gel with controlled density using dynamic imine bonds: pH-controlled release and extended duration. Carbohydr. Polym..

[55] Tang X., Zhu M., Zhang L., Zhu L. (2025). Development of cinnamaldehyde/aminated gelatin film as pH-responsive controlled-release packaging for cherry preservation: Effects of CO_2_ and humidity in the microenvironment. Food Packag. Shelf Life.

[56] Liu Y., Chang J., Mao J., Wang S., Guo Z., Hu Y. (2023). Dual-network hydrogels based on dynamic imine and borate ester bonds with antibacterial and self-healing properties. Colloids Surf. B Biointerfaces.

[57] Mohammadzadeh A., Javanbakht S., Mohammadi R. (2024). Magnetic alginate core-shell nanoparticles based on Schiff-base imine bonding for pH-responsive doxorubicin delivery system. Colloids Surf. A Physicochem. Eng. Asp..

[58] Hao Y., Zhou M., Chen R., Mao X., Huang W.-C. (2023). A bioinspired hydrogel carrier with pH/redox dual responsiveness for effective protection and intestinal targeted delivery of probiotics. J. Food Eng..

[59] Qian Z., Rong L., Yuan W. (2024). pH and redox dually responsive micelles loaded with anti-cancer drug and OA-Bi2S3 nanodots for chemo-photothermal synergistic treatment of cancers. Mater. Lett..

[60] Shee M., Lal Banerjee S., Dey A., Das Jana I., Basak P., Mandal M., Mondal A., Kumar Das A., Das N.C. (2024). pH-induced fluorescent active sodium alginate-based ionically conjugated and REDOX responsive multi-functional microgels for the anticancer drug delivery. Int. J. Pharm..

[61] Ali A., Javed R., Farhangi S., Shah T., Ullah S., ul Ain N., Liu T., Guo Z., Lynch I., Raza F. (2023). Metal phenolic networks (MPNs)-based pH-sensitive stimulus responsive nanosystems for drug delivery in tumor microenvironment. J. Drug Deliv. Sci. Technol..

[62] Guo Z., Li H., Ma J., Xu G., Jia Q. (2024). pH-sensitive metal–organic framework carrier decorated with chitosan for controlled drug release. Int. J. Pharm..

[63] Liu S., Chen Y., Li X., Yao Y., Wang H., Wang M. (2025). pH-responsive starch-based bilayer film functionalized with alliin loaded MIL-101 (Fe) for active food packaging. Carbohydr. Polym..

[64] Cao L., Gong Z., Liu C., Fan J., Chen Y. (2021). Design and fabrication of mechanically strong and self-healing rubbers via metal-ligand coordination bonds as dynamic crosslinks. Compos. Sci. Technol..

[65] Zhu L., Wang D., Wei X., Zhu X., Li J., Tu C., Su Y., Wu J., Zhu B., Yan D. (2013). Multifunctional pH-sensitive superparamagnetic iron-oxide nanocomposites for targeted drug delivery and MR imaging. J. Control. Release.

[66] Li S., Dai W., Yin Z.-Z., Gao J., Wu D., Kong Y. (2020). Synthesis of oxidized pullulan coated mesoporous silica for pH-sensitive drug delivery. Eur. Polym. J..

[67] Zhou Z., Wang Z., Liu X., Zhao Z., An H., Wang Y., He Y., Qin J. (2022). Pectin-based self-healing hydrogel through acylhydrazone connection for controlled drug release and enhanced tumor therapy. J. Drug Deliv. Sci. Technol..

[68] Shi Y., Pan X., Xu S., Zhu H., Zhao B., Sun Z., Dong R., Li N., Hou X., Yang X. (2023). Synthesis of the pH-sensitive nanoparticles based on the acylhydrazone bonds conjugated doxorubicin and studies on their in vivo anti-tumor effects. Eur. J. Med. Chem..

[69] Cheng H., Fan Z., Wang Z., Guo Z., Jiang J., Xie Y. (2023). Highly stretchable, fast self-healing nanocellulose hydrogel combining borate ester bonds and acylhydrazone bonds. Int. J. Biol. Macromol..

[70] Lin C.X., Yang K., Li P.C., Gao L.T., Aziz Y., Li J.H., Miyatake H., Ito Y., Chen Y.M. (2024). Self-healing and injectable chitosan/konjac glucomannan hydrogel with pH response for controlled protein release. Colloids Surf. B Biointerfaces.

[71] Ren X., Wang S., Teng Y., Zheng S., Li F., Wang C., Wu L., Zhang J. (2025). Engineered extracellular vesicles loaded in boronated cyclodextrin framework for pulmonary delivery. Carbohydr. Polym..

[72] Chen J.-X., Shi Y., Zhang Y.-R., Teng L.-P., Chen J.-H. (2016). One-pot construction of boronate ester based pH-responsive micelle for combined cancer therapy. Colloids Surf. B Biointerfaces.

[73] Kim T.M., Subba S.H., Hwang Y.K., Kim S.G., Park J., Jin E.-J., Park S.Y. (2025). Electrical and fluorescence in situ monitoring of tumor microenvironment-based pH-responsive polymer dot coated surface. Talanta.

[74] Wang S., Nie F., Lin Z., Xu J., Guo Y. (2025). Natural polysaccharide-small molecule smart responsive nanogels: Design, synthesis, and synergistic chemoimmunotherapy for tumors. Int. J. Biol. Macromol..

[75] Liang S., Chen H., Chen Y., Ali A., Yao S. (2024). Multi-dynamic-bond cross-linked antibacterial and adhesive hydrogel based on boronated chitosan derivative and loaded with peptides from Periplaneta americana with on-demand removability. Int. J. Biol. Macromol..

[76] Altinbasak I., Kocak S., Sanyal R., Sanyal A. (2023). Redox-responsive nanogels for drug-delivery: Thiol–maleimide and thiol–disulfide exchange chemistry as orthogonal tools for fabrication and degradation. Polym. Chem..

[77] Marimuthu M., Arumugam S.S., Sabarinathan D., Li H., Chen Q. (2021). Metal organic framework based fluorescence sensor for detection of antibiotics. Trends Food Sci. Technol..

[78] Chen J., Wang C., Zhu Z.-Y., Wang F., Shang J., Liu Z., Wang L. (2024). Titanium-based metal-organic frameworks as pH-responsive drug delivery carriers of 5-Fluorouracil. J. Solid State Chem..

[79] Min T., Yue J., Cheng C., Ma X., Weng S., Luo Y., Lei Y., Long Y. (2024). ZIF-8/TOCNF carrier coated with pectin “gatekeeper” for pH/enzyme dual-responsive releasing of carvacrol and its preservation effect on fruits. Chem. Eng. J..

[80] Dwitya S.S., Lin K.-S., Weng M.-T., Mdlovu N.V., Lai L.-J., Wu C.-M. (2025). Thermo- and pH-responsive MOF-303 mediated P127 and Gelatin coating for combination drug release and liver cancer therapy. Mater. Today Chem..

[81] Wu Z., Chen Y., Peng Y., Xue H., Yao Y., Yang S., Pan C., Zhang D., Xie Y. (2025). Sodium-lignosulfonate-conjugated metal–organic frameworks as dual-stimulus-responsive carriers for improved pesticide targeting. Int. J. Biol. Macromol..

[82] Zhang Z., Zhang Y., Wang C., Liu X., El-Seedi H.R., Gómez P.L., Alzamora S.M., Zou X., Guo Z. (2024). Enhanced composite Co-MOF-derived sodium carboxymethyl cellulose visual films for real-time and in situ monitoring fresh-cut apple freshness. Food Hydrocoll..

[83] Pooresmaeil M., Namazi H. (2023). pH-sensitive carboxymethyl starch-gelatin coated COF/5-Fu for colon cancer therapy. Ind. Crops Prod..

[84] Fu D., Zhong L., Xu J., Mo A., Yang M. (2024). Hydrazone-functionalized nanoscale covalent organic frameworks as a nanocarrier for pH-responsive drug delivery enhanced anticancer activity. RSC Adv..

[85] Wang C., Qiao K., Ding Y., Liu Y., Niu J., Cao H. (2023). Enhanced control efficacy of spinosad on corn borer using polylactic acid encapsulated mesoporous silica nanoparticles as a smart delivery system. Int. J. Biol. Macromol..

[86] Wang D., Fan S., Li X., Chen L., Wen X., Xu Y., Zhu C., Hou C., Zhang D. (2024). Carboxymethyl chitosan/polyvinyl alcohol hydrogel films by incorporating MSNs as ε-PL carrier with pH-responsive controlled release and antibacterial properties. Food Packag. Shelf Life.

[87] Jiao T., Dong C., Zhu A., Ahmad W., Peng L., Wu X., Chen Q., Wei J., Chen X., Qin O. (2025). AFB1-responsive mesoporous silica nanoparticles for AFB1 quantification based on aptamer-regulated release of SERS reporter. Food Chem..

[88] Ding X., Ahmad W., Rong Y., Wu J., Ouyang Q., Chen Q. (2024). A dual-mode fluorescence and colorimetric sensing platform for efficient detection of ofloxacin in aquatic products using iron alkoxide nanozyme. Food Chem..

[89] Li Y., Zhao L., Bai Y., Feng F. (2025). Applications of covalent organic frameworks (COFs)-based sensors for food safety: Synthetic strategies, characteristics and current state-of-art. Food Chem..

[90] Chen T., Huang C., Ye C., Li L., Liu Z., Huang W., Lin L., Li C., Ye Y. (2023). Controlled release and antibacterial properties of nanofiber membrane loaded with tea saponin. Ind. Crops Prod..

[91] Guan B., Wang F., Jiang H., Zhou M., Lin H. (2022). Preparation of Mesoporous Silica Nanosphere-Doped Color-Sensitive Materials and Application in Monitoring the TVB-N of Oysters. Foods.

[92] Kassem A.M., Almukainzi M., Faris T.M., Ibrahim A.H., Anwar W., Elbahwy I.A., El-Gamal F.R., Zidan M.F., Akl M.A., Abd-ElGawad A.M. (2024). A pH-sensitive silica nanoparticles for colon-specific delivery and controlled release of catechin: Optimization of loading efficiency and in vitro release kinetics. Eur. J. Pharm. Sci..

[93] Sangwan P., Barala P., Hooda V.J.B., Biotechnology A. (2025). Enhancing melatonin photostability and controlled release using pH-responsive mesoporous silica nanoparticles for agricultural applications. Biocatal. Agric. Biotechnol..

[94] Chang J., Mo L., Song J., Wang X., Liu H., Meng C., Wu Y. (2022). A pH-responsive mesoporous silica nanoparticle-based drug delivery system for targeted breast cancer therapy. J. Mater. Chem. B.

[95] Li Q., Lai S., Shang H., Qiao N., Sun X., Lu Y., Wang Z., Wang X., Wu Y. (2024). Construction and evaluation of biomass-modified mesoporous silica nanoparticles as enzyme-responsive and pH-Responsive drug carriers for the controlled release of quercetin. J. Drug Deliv. Sci. Technol..

[96] Hu Z., Wang H., Li L., Wang Q., Jiang S., Chen M., Li X., Jiang S. (2021). pH-responsive antibacterial film based polyvinyl alcohol/poly (acrylic acid) incorporated with aminoethyl-phloretin and application to pork preservation. Food Res. Int..

[97] Li H., Wang C., Shi H. (2025). Development of endolysin-integrated pH-responsive antiadhesive and antibacterial coatings with nanorods for the prevention of cross-contamination in fresh produce. Food Res. Int..

[98] Arafat M.T., Mahmud M.M., Wong S.Y., Li X. (2021). PVA/PAA based electrospun nanofibers with pH-responsive color change using bromothymol blue and on-demand ciprofloxacin release properties. J. Drug Deliv. Sci. Technol..

[99] Huang X., Zhao W., Li Z., Zhang J., Zhang N., Shi J., Zhai X., Shen T., Zou X. (2024). pH-triggered bilayer film based on carboxymethyl cellulose/zein/Eudragit L100 with purple cabbage anthocyanin for monitoring pork freshness. Int. J. Biol. Macromol..

[100] Čalija B., Cekić N., Savić S., Daniels R., Marković B., Milić J. (2013). pH-sensitive microparticles for oral drug delivery based on alginate/oligochitosan/Eudragit^®^ L100-55 “sandwich” polyelectrolyte complex. Colloids Surf. B Biointerfaces.

[101] Khan M.Z.I., Prebeg Ž., Kurjaković N. (1999). A pH-dependent colon targeted oral drug delivery system using methacrylic acid copolymers: I. Manipulation of drug release using Eudragit^®^ L100-55 and Eudragit^®^ S100 combinations. J. Control. Release.

[102] Zeeshan M., Ali H., Khan S., Khan S.A., Weigmann B. (2019). Advances in orally-delivered pH-sensitive nanocarrier systems; an optimistic approach for the treatment of inflammatory bowel disease. Int. J. Pharm..

[103] Dong P., Sahle F.F., Lohan S.B., Saeidpour S., Albrecht S., Teutloff C., Bodmeier R., Unbehauen M., Wolff C., Haag R. (2019). pH-sensitive Eudragit^®^ L 100 nanoparticles promote cutaneous penetration and drug release on the skin. J. Control. Release.

[104] Aguilar L.E., Unnithan A.R., Amarjargal A., Tiwari A.P., Hong S.T., Park C.H., Kim C.S. (2015). Electrospun polyurethane/Eudragit^®^ L100-55 composite mats for the pH dependent release of paclitaxel on duodenal stent cover application. Int. J. Pharm..

[105] Ji Q., Yu X., Wu P., Yagoub A.E.-G.A., Chen L., Abdullateef Taiye M., Zhou C. (2021). Pretreatment of sugarcane bagasse with deep eutectic solvents affect the structure and morphology of lignin. Ind. Crops Prod..

[106] Anushikha, Gaikwad K.K. (2023). Lignin as a UV blocking, antioxidant, and antimicrobial agent for food packaging applications. Biomass Convers. Biorefinery.

[107] Roy S., Priyadarshi R., Purohit S.D., Rhim J.-W. (2023). Antimicrobial and antioxidant properties of lignin and its composites. Lignin-Based Materials Health Care and Medical Applications.

[108] Yi C., Xu Q., Yang D., Wang M. (2022). A novel pH-responsive charge reversal nanospheres based on acetylated histidine-modified lignin for drug delivery. Ind. Crops Prod..

[109] Gao H., Seidi F., Cai Y., Sun Z., Bian H., Dai H., Xu T. (2025). Construction of curcumin-conjugated pH-responsive lignin-based nanoparticles for alleviating oxidative stress: Stability, antioxidant activity and biocompatibility. Int. J. Biol. Macromol..

[110] Yan S., Chai L., Li W., Xiao L.-P., Chen X., Sun R.-C. (2022). Tunning the properties of pH-responsive lignin-based hydrogels by regulating hydroxyl content. Colloids Surf. A Physicochem. Eng. Asp..

[111] Wang M., Yang D., Xu Q., Li P., Yi C., Qian Y., Qiu X. (2021). Highly efficient evaporation method to prepare pH-responsive lignin-hollow-nanosphere with controllable size and its application in oral drug delivery. Ind. Crops Prod..

[112] Shan Z., Jiang B., Wang P., Wu W., Jin Y. (2025). Sustainable lignin-based composite hydrogels for controlled drug release and self-healing in antimicrobial wound dressing. Int. J. Biol. Macromol..

[113] Kumar S., Cherwoo L., Puri N., Sharma A., Thombare N., Bhondekar A.P. (2023). Shellac: A natural lipid polymer for food safety and quality monitoring. Nanotechnology Applications for Food Safety and Quality Monitoring.

[114] Baek J., Ramasamy M., Cho D.G., Chung Soo C.C., Kapar S., Lee J.Y., Tam K.C. (2023). A new approach for the encapsulation of Saccharomyces cerevisiae using shellac and cellulose nanocrystals. Food Hydrocoll..

[115] Yin M., Zhang Q., Zhong F. (2024). Construction of double network gel for co-encapsulation of probiotics and capsaicin: Enhanced the physicochemical stability and controlled release. Food Biosci..

[116] Mu X., Roghzai H., Zeng L., Sun X., Zhao X. (2025). Curcumin-loaded zein and shellac composite nanoparticles for ulcerative colitis treatment. Eur. J. Pharm. Biopharm..

[117] Shu D., Liu Y., Xu J., Yuan Y. (2025). A review of shellac-based carrier design for food application: From the perspective of core materials. LWT.

[118] Cui H., Yang X., Li C., Ye Y., Chen X., Lin L. (2022). Enhancing anti-*E. coli* O157:H7 activity of composite phage nanofiber film by D-phenylalanine for food packaging. Int. J. Food Microbiol..

[119] Yang Z., Li M., Li Y., Huang X., Li Z., Zhai X., Shi J., Zou X., Xiao J., Sun Y. (2024). Sodium alginate/guar gum based nanocomposite film incorporating β-Cyclodextrin/persimmon pectin-stabilized baobab seed oil Pickering emulsion for mushroom preservation. Food Chem..

[120] Tsai F.-H., Chiang P.-Y., Kitamura Y., Kokawa M., Islam M.Z. (2017). Producing liquid-core hydrogel beads by reverse spherification: Effect of secondary gelation on physical properties and release characteristics. Food Hydrocoll..

[121] Tan Y., Chan S., Wu B., Wang H., Lou Z. (2025). Fabrication of pH-responsive whey protein/sodium alginate composite hydrogel beads for theaflavins. J. Food Eng..

[122] Ma L., Long W., Liu Y. (2025). The fabrication of gastrointestinal pH-responsive sodium alginate surface-modified protein vehicles to improve limonin digestive stability, bioaccessibility and trans-intestinal mucus barrier capacity. Food Hydrocoll..

[123] Truong-Le Q.-A., Lee S.-O., Ubeyitogullari A. (2025). Encapsulation of Bifidobacterium bifidum into a pH-sensitive alginate-pectin gel system using 3D food printing: Enhanced viability and targeted release. Int. J. Biol. Macromol..

[124] Ren J., Jin J., Wang Y., Liu Y., Qi H. (2025). Preparation and drug release behavior study of intelligent pH-Responsive sodium alginate/κ-carrageenan polysaccharide-based hydrogels. Food Biosci..

[125] Iqbal M.W., Riaz T., Yasmin I., Leghari A.A., Amin S., Bilal M., Qi X. (2021). Chitosan-Based Materials as Edible Coating of Cheese: A Review. Starch-Stärke.

[126] Cui H., Surendhiran D., Li C., Lin L. (2020). Biodegradable zein active film containing chitosan nanoparticle encapsulated with pomegranate peel extract for food packaging. Food Packag. Shelf Life.

[127] Ji Q., Su L., Boateng I.D., Li Z., Zhou C., Liu X., Ma Y. (2025). Preparation of chitosan/peanut shell nano-lignocellulose (CS/NLC) composite film and its preservation effect on cherry tomato and blueberry. Ind. Crops Prod..

[128] Jiao X., Chong X., Du H., Yang M., Zhu Z., Ma Z., Wen Y. (2025). Development of pH and enzyme dual responsive chitosan/polyaspartic acid nanoparticle-embedded nanofibers for fruit preservation. Int. J. Biol. Macromol..

[129] Liu H., Huang Z., Shi Y., Cai T., Miao Q., Gao Z., Cui Z. (2024). Lightweight pH-responsive chitosan hydrogel iron fertilizer: Efficient performance, controlled-release, and tomato application. J. Environ. Chem. Eng..

[130] Anees Ur Rehman Qureshi M., Arshad N., Rasool A., Rizwan M., Fahmi Fawy K., Rasheed T. (2024). pH-responsive chitosan dendrimer hydrogels enabling controlled cefixime release. Eur. Polym. J..

[131] Pourmadadi M., Abdouss H., Memarzadeh A., Abdouss M., Fathi-karkan S., Rahdar A., Díez-Pascual A.M. (2024). Innovative chitosan-polyacrylic acid-MoS2 nanocomposite for enhanced and pH-responsive quercetin delivery. Mater. Today Commun..

[132] Su Y., Sun M., Zhao M., Xu B., Li J., Zheng T. (2022). Enhancement of the physicochemical and in vitro release properties of lutein by gelatin/octenyl succinic anhydride (OSA)-modified starch composite as vehicles. Int. J. Food Sci. Technol..

[133] Li D., Liu A., Liu M., Li X., Guo H., Zuo C., Li Y. (2020). The intestine-responsive lysozyme nanoparticles-in-oxidized starch microgels with mucoadhesive and penetrating properties for improved epithelium absorption of quercetin. Food Hydrocoll..

[134] Wu C.-N., Lai H.-M. (2019). Novel pH-responsive granules with tunable volumes from oxidized corn starches. Carbohydr. Polym..

[135] Ekumah J.-N., Adade S.Y.-S.S., Nunekpeku X., Zhong M., Sun Y., Liang Q., Virk M.S., Johnson N.A.N., Kwadzokpui B.A., Ren X. (2025). Nondestructive prediction and classification of gel strength in ethanol-treated kudzu starch gels using near-infrared spectroscopy. Food Chem..

[136] Zhang Z.-H., Li M.-F., Peng F., Zhong S.-R., Huang Z., Zong M.-H., Lou W.-Y. (2021). Oxidized high-amylose starch macrogel as a novel delivery vehicle for probiotic and bioactive substances. Food Hydrocoll..

[137] Zhu J., Xie F., Qiu Z., Chen L. (2024). Effect of active carbonyl-carboxyl ratio on dynamic Schiff base crosslinking and its modulation of high-performance oxidized starch-chitosan hydrogel by hot extrusion 3D printing. Carbohydr. Polym..

[138] León O., Soto D., Antúnez A., Fernández R., González J., Piña C., Muñoz-Bonilla A., Fernandez-García M. (2019). Hydrogels based on oxidized starches from different botanical sources for release of fertilizers. Int. J. Biol. Macromol..

[139] Zhang H., Liang S., Quintero L.E.E., Wang Q., Chen S., Gong Y., Liu B., Yuan Y., Li Y. (2022). The structure design and application of oxidized polysaccharides delivery systems for controlled uptake and release of food functional ingredients. Curr. Opin. Food Sci..

[140] Xie F., De Wever P., Fardim P., Van den Mooter G. (2021). TEMPO-oxidized cellulose beads as potential pH-responsive carriers for site-specific drug delivery in the gastrointestinal tract. Molecules.

[141] Lin X., Guo L., Shaghaleh H., Hamoud Y.A., Xu X., Liu H. (2021). A TEMPO-oxidized cellulose nanofibers/MOFs hydrogel with temperature and pH responsiveness for fertilizers slow-release. Int. J. Biol. Macromol..

[142] Tian F., Xu S., Gan M., Chen B., Luan Q., Cai L. (2025). Bionic cell wall models: Utilizing TEMPO-oxidized cellulose nanofibers for fucoxanthin delivery systems. Carbohydr. Polym..

[143] Dabbour M., Jiang H., Mintah B.K., Wahia H., He R. (2021). Ultrasonic-assisted protein extraction from sunflower meal: Kinetic modeling, functional, and structural traits. Innov. Food Sci. Emerg. Technol..

[144] Javed M., Huang H., Ma Y., Ettoumi F.-e., Wang L., Xu Y., El-Seedi H.R., Ru Q., Luo Z. (2024). Construction of self-assembled nano cellulose crystals/chitosan nanobubbles composite hydrogel with improved gallic acid release property. Food Chem..

[145] Ebadi A., Rafati A.A., Bavafa S., Mohammadi M. (2017). Kinetic and theoretical studies of novel biodegradable thermo-sensitive xerogels based on PEG/PVP/silica for sustained release of enrofloxacin. Appl. Surf. Sci..

[146] Aycan D. (2024). Alginate/hyaluronic acid/gelatin ternary blended films as pH-sensitive drug carriers: In vitro ampicillin release and kinetic studies. Int. J. Biol. Macromol..

[147] Aslehashemi A., Miralinaghi M., Heydarinasab A. (2025). A magnetic/pH dual-sensitive nanocarrier based on biopolymer-grafted mesoporous Fe_3_O_4_@SiO_2_ for imatinib delivery: Fabrication, characterization, and study of the in-vitro release kinetics. Inorg. Chem. Commun..

[148] Jafari H., Namazi H. (2024). Chitosan/laponite clay bio-nanocomposite as an efficient cytocompatible nanocarrier for pH-sensitive controlled release of curcumin. Inorg. Chem. Commun..

[149] Liu Y., Kang W., Nie L., Xiao F., Li Y., Ma Q., Lin D., Zhou G., Liu S., Sun K. (2024). Alginate/methacrylic acid/calcium ion-based pH-sensitive drug delivery hydrogel for the treatment of ulcerative colitis. React. Funct. Polym..

[150] Gong Y., Zhang Q., Yuan H., Luo X. (2025). Fabrication of carboxylated cellulose microspheres via Fe^2+^/H_2_O_2_ oxidation method and their application in vitamin B12 efficient loading and controlled release. J. Drug Deliv. Sci. Technol..

[151] Nath J., Ahmed A., Saikia P., Chowdhury A., Dolui S.K. (2020). Acrylic acid grafted gelatin/LDH based biocompatible hydrogel with pH-controllable release of vitamin B12. Appl. Clay Sci..

[152] Arjeh E., Rostami H., Pirsa S., Chopani A., Ahmadi A., Fathi M. (2024). Facile and green fabrication of algal protein/M^(n+)^ hydrogel through coordination with cations for sustained release of vitamin B6. Algal Res..

[153] Zhou X., Feng X., Qi W., Zhang J., Chen L. (2024). Microencapsulation of vitamin E by gelatin-high/low methoxy pectin complex coacervates: Effect of pH, pectin type, and protein/polysaccharide ratio. Food Hydrocoll..

[154] Yang Q., Wang Y.-R., Liu Q.-Q., Chen H.-Q. (2023). Development of arachin and basil seed gum composite gels for the encapsulation and controlled release of vitamin D3. Int. J. Biol. Macromol..

[155] Srivastava N., Choudhury A.R. (2023). Enhanced encapsulation efficiency and controlled release of co-encapsulated Bacillus coagulans spores and vitamin B9 in gellan/κ-carrageenan/chitosan tri-composite hydrogel. Int. J. Biol. Macromol..

[156] Tao R., Li G., Wang S., Sun Y., Li Y., Wang P., Huang S., Fan B., Wang F. (2025). Natural, tough and pH-responsive ultrasound modified soy protein isolate-arabinoxylan based double network hydrogels for controlled nutrients release. Food Hydrocoll..

[157] Xu R., Deng W., Dai Y., Hu J. (2022). pH-responsive citral microcapsules with tannic acid-FeIII coordination complexes. Food Chem..

[158] Yu W., You H., Li X., Wang H., Xu J., Chen H., Sun B., Wang X. (2024). pH-responsive chitosan hollow microspheres pro-flavor based on interfacial Schiff-base bonding for controlled release of cinnamaldehyde. Food Hydrocoll..

[159] Li Z., Liu T., Fan K., Geng L., Wang P., Ren F., Luo J. (2024). Preparation of pH-responsive chitosan microspheres containing aminopeptidase and their application in accelerating cheese ripening. J. Dairy Sci..

[160] Cao Y., Dong Y., Wu T., Chen L., Zhu W., Jiang T., He N., Liu Y., Huang R., Yu X. (2025). A carboxymethyl cellulose-based pH-responsive chlorine dioxide release film for strawberry preservation. Int. J. Biol. Macromol..

[161] Sun H., Qiu X., Li X., Wang H. (2024). Eco-friendly, pH-sensitive curcumin-loaded sodium alginate/hydroxyapatite/quaternary ammonium chitosan microspheres with enhanced antibacterial and antioxidant activities for fruit preservation. Int. J. Biol. Macromol..

[162] Ma Y., Zhang X., Li J., Bi Z., Li J., Ma Z., Huang H., Li Y. (2025). Development of electrospun nanofiber membranes based on ZIF-8 MOF loaded tea polyphenols with pH-responsive release capabilities for food preservation. Innov. Food Sci. Emerg. Technol..

[163] Li Q., Ren T., Perkins P. (2022). The development and application of nanocomposites with pH-sensitive “gates” to control the release of active agents: Extending the shelf-life of fresh wheat noodles. Food Control.

[164] Li L., Xu Z., Yang H., Zhao W., Tao Y., Lu J., Xia X., Tan M., Du J., Wang H. (2025). pH triggered on-demand tannic acid release via “open-close” of soybean isolate proteins spatial structure for shrimp preservation. Food Control.

[165] Zhang Y., Cheng X., Guo C., Lu L., Lu L., Qiao Y. (2025). pH-responsive controlled-release antimicrobial active film based on gelatin/chitosan incorporated with HNT nanocomposite. Chem. Eng. J..

[166] Shaghaleh H., Hamoud Y.A., Xu X., Liu H., Wang S., Sheteiwy M., Dong F., Guo L., Qian Y., Li P. (2021). Thermo-/pH-responsive preservative delivery based on TEMPO cellulose nanofiber/cationic copolymer hydrogel film in fruit packaging. Int. J. Biol. Macromol..

[167] Wang B., Qiu D., Gu Y., Shan Z., Shi R., Luo J., Qi S., Wang Y., Jiang B., Jin Y. (2025). A lignin-based controlled/sustained release hydrogel by integrating mechanical strengthening and bioactivities of lignin. J. Bioresour. Bioprod..

[168] Fatema K.N., Li L., Ahmad K., Kim J., Lee D.-W. (2025). Development of multifunctional PAA-alginate-carboxymethyl cellulose hydrogel-loaded fiber-reinforced biomimetic scaffolds for controlled release of curcumin. Int. J. Biol. Macromol..

